# Mitochondrial‐Derived Peptide MOTS‐c Suppresses Ovarian Cancer Progression by Attenuating USP7‐Mediated LARS1 Deubiquitination

**DOI:** 10.1002/advs.202405620

**Published:** 2024-09-25

**Authors:** Yadong Yin, Yujie Li, Boyi Ma, Chenlu Ren, Shuhua Zhao, Jia Li, Yun Gong, Hong Yang, Jibin Li

**Affiliations:** ^1^ Department of Gynaecology and Obstetrics Xijing Hospital Air Force Medical University Xi'an 710032 China; ^2^ Department of Traditional Chinese Medicine The First Affiliated Hospital of Xi'an Jiaotong University Xi'an 710061 China; ^3^ State Key Laboratory of Holistic Integrative Management of Gastrointestinal Cancers and Department of Physiology and Pathophysiology Air Force Medical University Xi'an 710032 China

**Keywords:** LARS1, MOTS‐c, ovarian cancer, ubiquitination, USP7

## Abstract

Mitochondrial‐nuclear communication plays a vital role in maintaining cellular homeostasis. MOTS‐c, a short peptide derived from the 12S rRNA of mitochondrial DNA, has been suggested as a retrograde mitochondrial signal. Although recent clinical studies have suggested a possible link between MOTS‐c and human cancer, the role of MOTS‐c in tumorigenesis has yet to be investigated. Here, MOTS‐c levels are found to be reduced in both serum and tumor tissues from ovarian cancer (OC) patients, which are associated with poor patients’ prognosis. Exogenous MOTS‐c inhibits the proliferation, migration and invasion of OC cells, and induces cell cycle arrest and apoptosis. Mechanistically, MOTS‐c interacts with LARS1 and promotes its ubiquitination and proteasomal degradation. In addition, USP7 was identified as a deubiquitinase of LARS1, and MOTS‐c can attenuates USP7‐mediated LARS1 deubiquitination by competing with USP7 for binding to LARS1. Besides, LARS1 was found to be increased and play an important oncogenic function in OC. More importantly, MOTS‐c displays a marked anti‐tumor effect on OC growth without systemic toxicity in vivo. In conclusion, this study reveals a crucial role of MOTS‐c in OC and provides a possibility for MOTS‐c as a therapeutic target for the treatment of this manlignacy.

## Graphical abstract

MOTS‐c, a peptide encoded by mitochondrial 12S rRNA, its expression is reduced in ovarian cancer, and a low MOTS‐c level is associated with poor prognosis. Exogenous MOTS‐c can inhibit OC cell growth in vitro and in vivo. Mechanistically, MOTS‐c exerts anti‐cancer effects by attenuating USP7‐mediated deubiquitination of LARS1 and promoting LARS1 degradation.

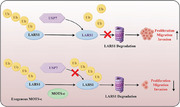

## 1 Introduction

Ovarian cancer (OC) is the deadliest gynecological malignancy, with 314 000 new diagnoses worldwide in 2020 and 207 000 deaths in the same year.^[^ ^1^ ^]^ Despite the temporary clinical remission achieved in OC patients through surgery and chemotherapy, the available treatment options are still limited, particularly for those facing tumor recurrence and resistance to chemotherapeutic drugs. As a result, the overall survival rate for individuals with OC has not shown substantial enhancement in the last three decades.^[^ ^2^ ^]^ Only 30.8% of patients with advanced OC survive for 5 years.^[^ ^3^ ^]^ Therefore, there is an urgent need to find new anti‐tumor targets and their therapeutic agents to prolong the survival of OC patients.

Mitochondria are bioenergetic and biosynthetic organelles. Cancer cells sustain their proliferation by reprogramming mitochondrial metabolism to provide raw materials for lipid, nucleic acid, and protein synthesis.^[^ ^4^ ^]^ In addition, mitochondria are also important hubs for intracellular communication and signal transduction. Under metabolic stress, mitochondria alter nuclear gene expression by generating a variety of retrograde signals, which help the cell adapt to an impaired homeostatic state.^[^ ^5^ ^]^ The involvement of mitochondrial‐nuclear communication is frequently observed in tumor cells. Therefore, targeting these mitochondrial retrograde signals (mitochondrial ROS, oncometabolites, mitochondrial calcium, and more) is currently a promising anti‐cancer strategy.^[^ ^5^, ^6^ ^]^

Recently, MOTS‐c, a mitochondria‐derived 16‐amino acid peptide encoded by mitochondrial 12S rRNA, has been recognized as a crucial factor involved in mitochondrial retrograde signaling regulation.^[^ ^7^ ^]^ Upon metabolic stress, MOTS‐c translocates to the nucleus and binds to the transcription factor NRF2 to participate in the regulation of nuclear gene expression and the maintenance of cellular homeostasis.^[^ ^7a^ ^]^ More recently, MOTS‐c was reported to be reduced in patients with hepatitis B virus‐associated hepatocellular carcinoma, and exogenous MOTS‐c treatment protected liver function by inhibiting hepatitis B virus DNA replication through promoting mitochondrial fusion.^[^ ^8^ ^]^ In addition, a significant increase in MOTS‐c levels was found in individuals with lung cancer after radiation therapy.^[^ ^9^ ^]^ Furthermore, MOTS‐c was associated with the likelihood of developing prostate and breast cancer, with differences by race.^[^ ^10^ ^]^ All of these studies indicate a probable link between MOTS‐c and tumor development. However, the biological functions of MOTS‐c remain largely unexplored in human cancers, including OC.

In this study, MOTS‐c levels were found to be reduced in OC and low MOTS‐c expression was associated with poor prognosis, indicating a potentially important role of MOTS‐c in the development of OC. Exogenous MOTS‐c was able to suppress tumor growth in vivo and in vitro. Mechanistically, MOTS‐c interacted with LARS1 and promoted its ubiquitination and proteasomal degradation. Further studies have demonstrated that USP7 acted as a deubiquitinase and stabilizer of LARS1, while MOTS‐c attenuated USP7‐mediated LARS1 deubiquitination by competing with USP7 for binding LARS1, leading to its degradation. Furthermore, we found a significantly high expression and important oncogenic function of LARS1 in OC. Overall, our findings provide new options for OC therapy.

## 2 Results

### 2.1 MOTS‐c Is Decreased in OC and its Downregulation Is Associated with Poor Patients’ Survival

**Figure 1:**
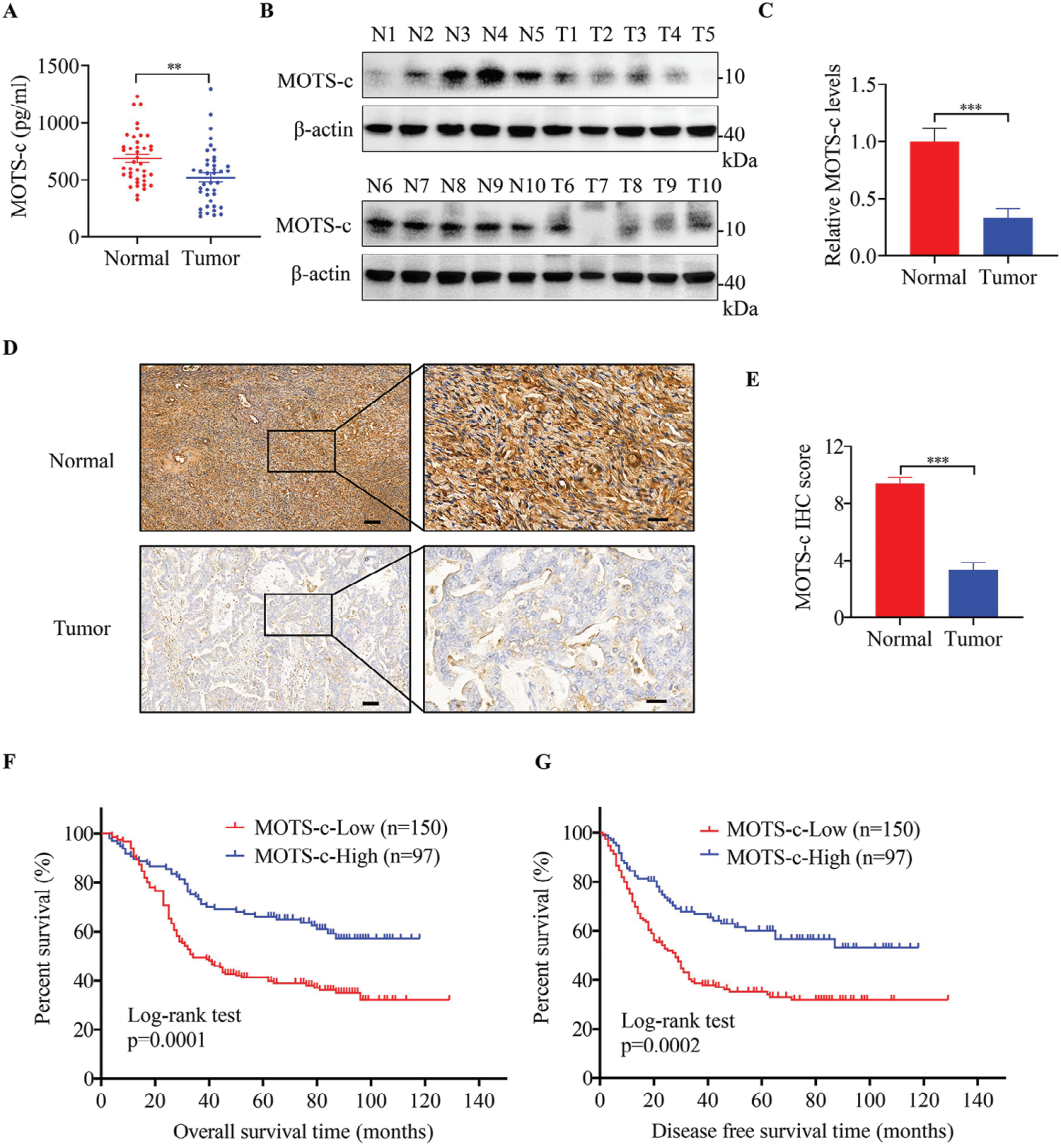
MOTS‐c is decreased in OC and its downregulation is associated with poor patients’ survival. A) ELISA assay was used to analyze serum MOTS‐c levels in healthy control women and OC patients (n = 40). B,C) Western blot analysis of MOTS‐c protein expression levels in OC tissues and adjacent normal tissues (n = 10). D,E) Immunohistochemistry (IHC) analysis of MOTS‐c expression levels in OC tissues and adjacent normal tissues (n = 247). Scale bars, 100 and 20 µm. F) Overall and G) disease‐free survival rates of OC patients with high and low MOTS‐c expression based on IHC scores were evaluated by Kaplan–Meier analysis. Data are presented as the mean ± SEM, ^**^ *p* < 0.01, ^***^ *p* < 0.001.

To investigate the role of MOTS‐c in OC, we first detected the levels of MOTS‐c in serum samples from 40 OC patients and 40 healthy women using an ELISA assay. The results showed that the levels of MOTS‐c were significantly lower in OC patients than in the healthy controls (**Figure** **1**A). Next, we determined the abundance of MOTS‐c in tumor tissues from 10 patients by Western blot assay, and found that MOTS‐c was significantly downregulated in OC tissues compared with adjacent normal tissues (Figure 1B,C). To validate this, we further analyzed the expression levels of MOTS‐c in 247 pairs of OC tissues and adjacent normal tissues by immunohistochemistry (IHC) staining assay. The results again showed that the levels of MOTS‐c in OC tissues were markedly decreased (Figure 1D,E). Additionally, Kaplan–Meier survival analyses showed that patients with low MOTS‐c expression had shorter overall and disease‐free survival (Figure 1F,G, respectively), and low levels of MOTS‐c were associated with higher clinical stage and greater susceptibility to metastasis (Table S1, Supporting Information). Together, these findings indicate that downregulation of MOTS‐c may play a crucial tumor‐promotive role during OC progression.

### 2.2 MOTS‐c Is Decreased in OC and its Downregulation Is Associated with Poor Patients’ Survival

**Figure 2:**
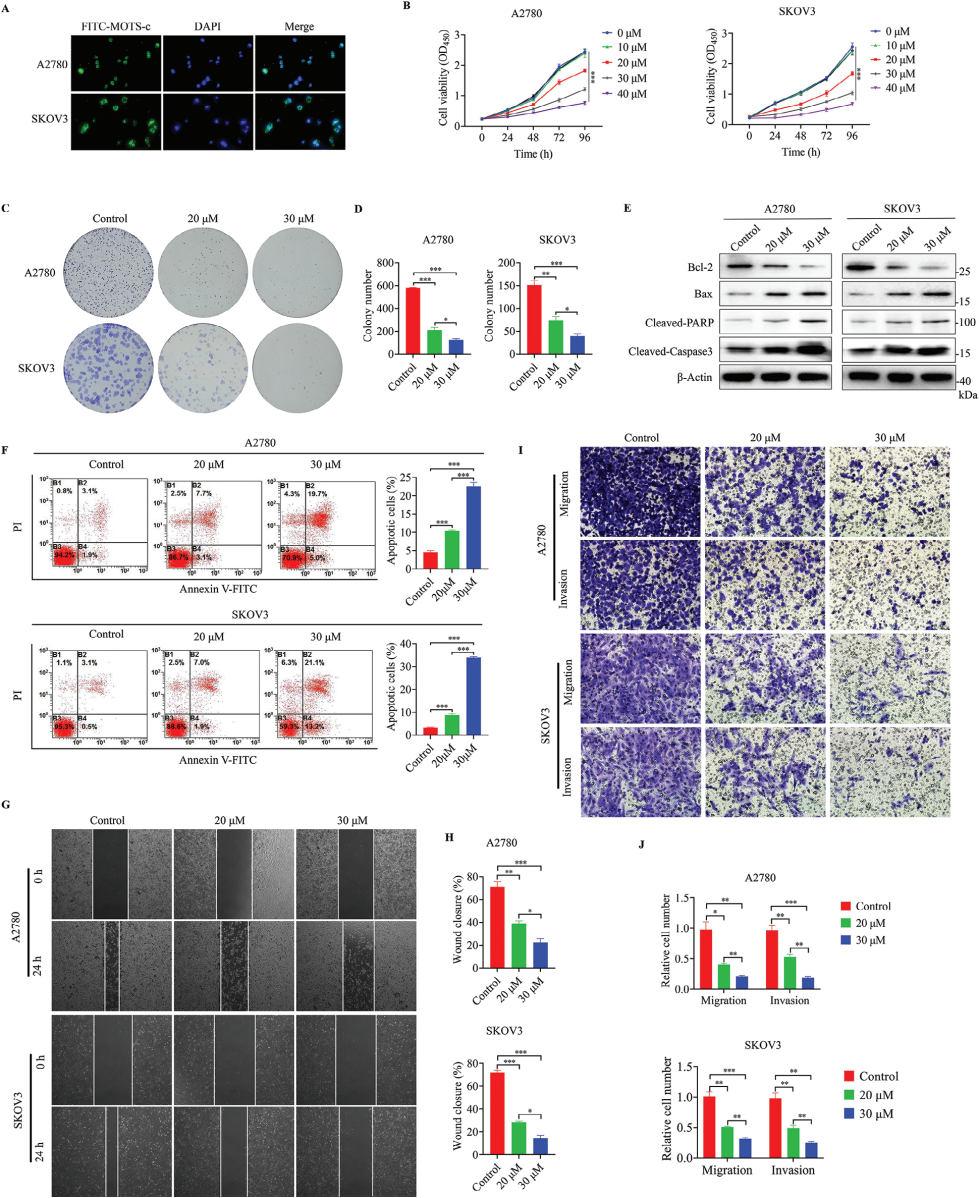
MOTS‐c inhibits the malignant progression of OC. A) Cellular uptake of MOTS‐c. A2780 and SKOV3 cells were incubated with 10 µm FITC‐MOTS‐c (green) at 37 °C for 3 h. Cell nuclei were stained with DAPI (blue) before imaging. B) CCK8 assay was performed in A2780 and SKOV3 cells treated with different concentrations of MOTS‐c (0, 10, 20, 30 and 40 µm). C,D) Colony formation assay was performed in A2780 and SKOV3 cells treated with 20 and 30 µm MOTS‐c E) Western blot assay was conducted for analyzing the expression of apoptosis‐related molecules BCL‐2, Bax, Cleaved‐PARP, and Cleaved‐Caspase3 in A2780 and SKOV3 cells treated with 20 and 30 µm MOTS‐c F) Flow cytometry assay was conducted to detect apoptosis levels in OC cells treated with 20 and 30 µm MOTS‐c. G,H) Wound healing and I,J) transwell assays assay were conducted in A2780 and SKOV3 cells treated with 20 and 30 µm MOTS‐c. Data are presented as the mean ± SEM, ^*^ *p* < 0.05, ^**^ *p* < 0.01, ^***^ *p* < 0.001.

Next, to evaluate the function of MOTS‐c in OC, we first examined the effective uptake of MOTS‐c in OC cells. FITC‐labeled MOTS‐c was synthesized and added to the culture medium, incubated with A2780 and SKOV3 cells for 3 h, and we found that FITC‐MOTS‐c was able to permeate the cell membrane and reach the cytoplasm and nucleus (**Figure** **2**A). Using the CCK8 assay (Figure 2B), MOTS‐c was found to be able to effectively inhibit the proliferation of A2780 and SKOV3 cells at concentrations exceeding 20 µm. The inhibitory effect was particularly evident at 30 µm, which prompted us to select 20 and 30 µm for further investigation. Colony formation assays showed that MOTS‐c markedly suppressed the proliferation of OC cells in a dose‐dependent manner (Figure 2C,D). Flow cytometry revealed that MOTS‐c treatment increased the apoptosis rate of OC cells (Figure 2F). Consistently, when apoptosis‐related molecules were analyzed by Western bot assay, we found that MOTS‐c treatment downregulated the expression of the anti‐apoptotic marker BCL‐2, while upregulated the expression of apoptotic markers Bax, Cleaved‐PARP, and Cleaved‐Capase3 (Figure 2E). In addition, cell‐cycle analysis revealed that MOTS‐c treatment induced S‐phase cell cycle arrest in OC cells (Figure S1A, Supporting Information). Moreover, wound healing (Figure 2G,H) and transwell assays (Figure 2I,J) indicated that MOTS‐c treatment weakened the migration and invasion capacities of OC cells. In conclusion, the above results suggested that MOTS‐c could effectively inhibit the malignant progression of OC cells.

### 2.3 MOTS‐c Interacts with and Promotes LARS1 Ubiquitination and Degradation

**Figure 3:**
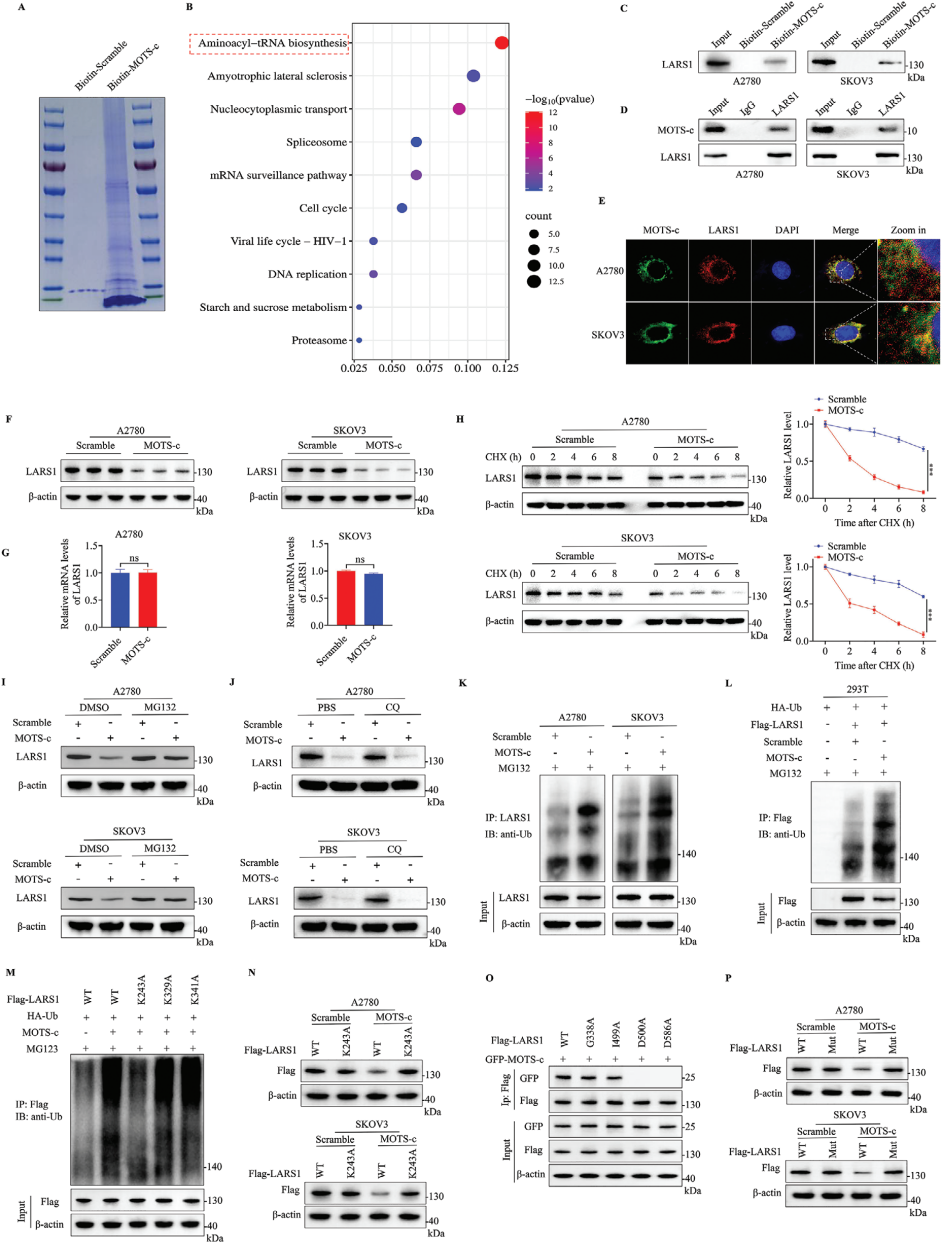
MOTS‐c promotes ubiquitination and proteasomal degradation of LARS1. A) A2780 cell lysate was pulled down with Biotin‐MOTS‐c, a scrambled‐sequence peptide (Biotin‐Scramble) was employed as a control. Lysates were separated by SDS‐PAGE and stained with Coomassie blue. B) MOTS‐c‐bound proteins were identified by mass spectrum (MS) and subjected to KEGG enrichment analysis. C) Western blot analysis was used to observe exogenous MOTS‐c binding to endogenous LARS1 in OC cells. D) Co‐immunoprecipitation (Co‐IP) assay was conducted to confirm the interaction of endogenous LARS1 and MOTS‐c in OC cells. E) Immunofluorescence (IF) assay was conducted for intracellular co‐localization of LARS1 and MOTS‐c proteins in OC cells. F) Western blot and G) RT‐qPCR assays were conducted for analyzing the expression of LARS1 in OC cells treated with 30 µm MOTS‐c. H) The stability of LARS1 protein was analyzed by Western blot, OC cells were treated with 100 µg mL^−1^ cycloheximide (CHX) for 0, 2, 4, 6, and 8 h before harvesting. I,J) OC cells were first treated with MOTS‐c, (I) 20 µm MG132 or (J) 50 µm chloroquine (CQ) were then added 8 h before harvesting, LARS1 levels were detected by Western blot. K) IP and immunoblot (IB) assays were conducted to analyze the ubiquitination levels of endogenous LARS1 in OC cells after MOTS‐c treatment, and MG132 was added 8 h before harvesting. L) IP and IB assays were performed to analyze the ubiquitination levels of exogenous LARS1 in MOTS‐c‐treated 293T cells transfected with HA‐Ub and Flag‐LARS1 plasmids. M) IP and IB assays were performed to confirm the ubiquitination site. Individual lysine residues mutated LARS1 (K243A, K329A, and K341A) and HA‐Ub were overexpressed in 239T cells. Cells were first treated with MOTS‐c, 20 µm MG132 was then added 8 h before harvesting. N) Western blot assays were conducted for analyzing the LARS1 protein levels in OC cells transfected with mutated LARS1 (K243A) and WT LARS1. The two groups of cells were treated with scramble or MOTS‐c, respectively. O) IP and IB assays were used to confirm the LARS1 binding sites. Individual amino acid residues mutated LARS1 (G338A, I499A, D500A, and D586A) and GFP‐MOTS‐c were overexpressed in 239T cells. P) Western blot assays were conducted for analyzing the LARS1 protein levels in OC cells transfected with mutated LARS1 (D500A & D586A) and WT LARS1. The two groups of cells were treated with scramble or MOTS‐c, respectively. Data are presented as the mean ± SEM, ^***^ *p* < 0.001.

Previous findings indicated that MOTS‐c suppresses the aggressive characteristics of OC cells. Nevertheless, the underlying mechanism remains a mystery. To find out, biotin‐tagged MOTS‐c (Biotin‐MOTS‐c) was synthesized for pull‐down assays, and mass spectrometry (MS) was used to analyze the binding proteins of MOTS‐c (**Figure** **3**A). Enrichment analysis of the MS data revealed a strong association between MOTS‐c and aminoacyl‐tRNA synthesis (AARS), as shown in Figure 3B. AARS plays critical roles in maintaining cell survival and tumor progression by catalyzing the binding of specific amino acids to their respective tRNAs and activating protein translation and synthesis.^[^ ^11^ ^]^ Our attention was focused on the top‐scoring protein, LARS1 (Figure S2A,B, Supporting Information), which is responsible for attaching leucine to its cognate tRNAs and serves as a leucine sensor to trigger mTORC1 activation.^[^ ^12^ ^]^ Knockdown or inhibition of LARS1 can effectively suppress tumor progression.^[^ ^13^ ^]^ Therefore, targeting LARS1 might be helpful in the treatment of OC. Next, we performed a Western blot with the protein mixtures obtained from the biotin pulldown assay, and found that exogenous MOTS‐c could bind to endogenous LARS1 (Figure 3C). Co‐immunoprecipitation (Co‐IP) assays further confirmed the interaction between endogenous LARS1 and MOTS‐c (Figure 3D). The intracellular co‐localization of MOTS‐c and LARS1 was confirmed by immunofluorescence (IF) staining (Figure 3E).

Next, we explored how MOTS‐c functions after binding with LARS1. Using Western blot and RT‐qPCR assays, we found that MOTS‐c treatment downregulated the protein level of LARS1 in OC cells (Figure 3F), but had no impact on mRNA level (Figure 3G), indicating that MOTS‐c may regulate LARS1 expression at the post‐transcriptional level. Therefore, we investigated whether MOTS‐c affected the stability of LARS1 protein using cycloheximide (CHX), and found that MOTS‐c administration notably shortened the half‐life of LARS1 protein in A2780 and SKOV3 cells (Figure 3H).

Then, we investigated how MOTS‐c facilitates LARS1 protein degradation. A2780 and SKOV3 cells were treated with MOTS‐c and then with the proteasome inhibitor MG132 or the lysosomal inhibitor chloroquine (CQ) for 8 h before harvesting. Western blot analysis showed that MOTS‐c‐mediated degradation of LARS1 was inhibited by MG132 (Figure 3I) but not by CQ (Figure 3J), suggesting that MOTS‐c enhances the degradation of LARS1 via a proteasome‐dependent pathway. Afterward, the impact of MOTS‐c on the ubiquitination of LARS1 was analyzed by IP and immunoblot (IB). The results showed a significant increase in the ubiquitination of both endogenous (Figure 3K) and exogenous (Figure 3L) LARS1 after treatment with MOTS‐c.

We next sought to determine the type of polyubiquitin chains that MOTS‐c promotes on LARS1. Wild‐type ubiquitin (HA‐Ub (WT)) and single lysine residue‐only ubiquitin (K6‐, K11‐, K27‐, K29‐, K33‐, K48‐, and K63‐ubiquitin) were overexpressed in 293 T cells. IP and IB assays showed that only K48‐ubiquitin promoted MOTS‐c mediated LARS1 ubiquitination (Figure S3A, Supporting Information). Meanwhile, we mutated each of the lysine residues on ubiquitin (K to R) to investigate its effect on MOTS‐c mediated LARS1 polyubiquitination in 293 T cells (Figure S3B, Supporting Information). The results showed that K48R ubiquitin significantly reduced the formation of ubiquitin chains on LARS1. Next, the ubiquitination sites of LARS1 (K243, K329, and K341) were predicted by MusiteDeep (https://www.musite.net/) and GPS‐Uber (https://gpsuber.biocuckoo.cn/online.php).^[^ ^14^ ^]^ We mutated individual lysine residues in LARS1 (K243A, K329A, and K341A). The results showed that mutation of K243 significantly reduced ubiquitination of LARS1, and can not be degraded by MOTS‐c treatment (Figure 3M,N). Thus, our data identified MOTS‐c‐mediated formation of a K48‐linked polyubiquitin chain on the K243 residue of LARS1.

To further validate the in vivo interaction between MOTS‐c and LARS1 and to gain molecular insight into the interaction between these two proteins, full length and domain‐truncated mutants of LARS1 were generated and over‐expressed in 293T cells (Figure S3C, Supporting Information). IP and IB assays indicated that amino acid residues 256–605 of LARS1 were required for the association of LARS1 with MOTS‐c (Figure S3D, Supporting Information). To make a clearer understanding of the interaction between MOTS‐c and LARS1, the amino acid binding sites at LARS1 and MOTS‐c were predicted by molecular docking (Figure S3E, Supporting Information). The results showed that G338, I499, D500, and D586 located in the region 256–605 of LARS1 formed good hydrogen bonding sites with MOTS‐c. To pinpoint the binding site, we mutated individual amino acid residues (G338A, I499A, D500A, and D586A) in LARS1. IP and IB assays showed that D500A and D586A mutants failed to interact with MOTS‐c (Figure 3O). Importantly, when D500 and D586 were simultaneously mutated (D500A&D586A, Flag‐LARS1‐Mut), MOTS‐c could not promote the degradation of the mutant LARS1 protein, compared with that of the WT LARS1 (Figure 3P). These data illustrated that D500 and D586 at LARS1 controlled its binding to MOTS‐c.

To sum up, the above data indicates that MOTS‐c interacts with and promotes LARS1 degradation via the ubiquitin‐proteasome pathway.

### 2.4 MOTS‐c Increases the Ubiquitination Level of LARS1 by Impairing USP7‐Mediated Deubiquitination

**Figure 4:**
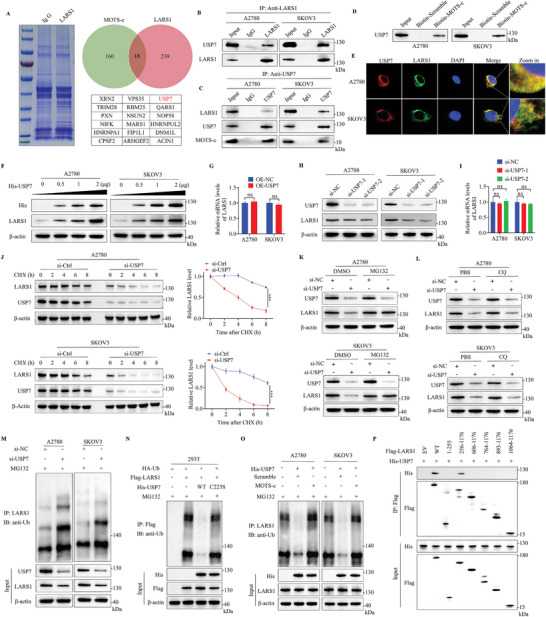
MOTS‐c increases the ubiquitination level of LARS1 by impairing USP7‐mediated deubiquitination of LARS1. A) A2780 cell lysate was immunoprecipitated by anti‐LARS1 antibody, the lysate was separated by SDS‐PAGE and stained with Coomassie blue. LARS1‐bound proteins were identified by MS and overlapped with proteins that bind to MOTS‐c. B,C) Co‐IP assays were conducted to observe the interaction between endogenous LARS1 and USP7, and USP7 binding to MOTS‐c in OC cells. D) Western blot assay was performed to observe exogenous MOTS‐c binding to endogenous USP7 in OC cells. E) IF assay was conducted for intracellular co‐localization of USP7 and LARS1 proteins in OC cells. F) Western blot assay was used to analyze LARS1 protein abundance in OC cells transfected with His‐USP7 plasmid (0.5, 1, 2 µg). G) RT‐qPCR assay was performed to analyze the mRNA level of LARS1 in OC cells transfected with USP7 overexpressing or empty plasmids. H) Western blot and I) RT‐qPCR assays were conducted for analyzing the expression of LARS1 in OC cells transfected with USP7 siRNA. J) The stability of LARS1 protein was analyzed by Western blot, OC cells were transfected with USP7 siRNA and treated with CHX before harvesting. K,L) OC cells were transfected with USP7 siRNA, (K) MG132, and (L) CQ were then added 8 h before harvesting, LARS1 levels were detected by Western blot. M) IP and IB assays were conducted to analyze the ubiquitination levels of endogenous LARS1 in OC cells transfected with USP7 siRNA. N) IP and IB assays were conducted to analyze the ubiquitination levels of exogenous LARS1 in 293T cells transfected with HA‐Ub, Flag‐LARS1, His‐USP7 WT, and His‐USP7 C223S plasmids. O) IP and IB assays were conducted to analyze the ubiquitination levels of endogenous LARS1 in OC cells transfected with His‐USP7 plasmid treated with 30 µM MOTS‐c or scramble peptide. P) IP and IB assays for testing the LARS1 domains critical for USP7 binding in 293T cells transfected with different structural domains of LARS1 plasmids and His‐USP7 plasmids. Data are presented as the mean ± SEM, ***p < 0.001.

We have already found that MOTS‐c can lead to LARS1 degradation via the ubiquitin‐proteasome pathway. However, the detailed mechanism remains to be elucidated. MOTS‐c does not belong to the family of ubiquitin ligases or deubiquitinating enzymes, so it is reasonable to hypothesize that there may be a ubiquitin ligase or deubiquitinating enzyme that plays a certain role between MOTS‐c and LARS1. Unfortunately, we did not find any molecules regulating LARS1 ubiquitination in our literature search. Therefore, we performed IP and MS analyses to identify proteins that bind to LARS1, and overlapped with proteins that bind to MOTS‐c. Finally, we obtained 18 proteins. Notably, USP7 was the only member of the deubiquitinating enzyme family among them (**Figure** **4**A; Figure S4A, Supporting Information). Then, we confirmed that USP7 and LARS1 could bind to each other, as well as USP7 and MOTS‐c (Figure 4B–D). The IF assay showed the co‐localization of USP7 and LARS1 in OC cells (Figure 4E).

Next, we investigated whether USP7 regulates LARS1 expression. We found that overexpression of USP7 upregulated the protein level of LARS1 in a dose‐dependent manner (Figure 4F), while leaving its mRNA level unaffected (Figure 4G). In contrast, knockdown of USP7 significantly downregulated the protein level of endogenous LARS1 in OC cells (Figure 4H), but again had no effect on its mRNA level (Figure 4I). Additionally, knockdown of USP7 markedly accelerated the rate of LARS1 degradation in OC cells exposed to CHX (Figure 4J). Furthermore, the destabilization of LARS1 induced by USP7 depletion could be reversed with MG132 (Figure 4K), but CQ did not have the same effect (Figure 4L). Next, we assessed the effect of USP7 on the ubiquitination level of LARS1. The results showed that knockdown of USP7 obviously increased the ubiquitination of endogenous LARS1 protein in OC cells (Figure 4M). In 293T cells cotransfected with USP7 and LARS1 overexpression plasmids, the ubiquitination of LARS1 induced by MG132 was significantly reduced by overexpressing the wild‐type USP7, but not the catalytically inactive mutant USP7 (C223S) (Figure 4N). These results suggest that USP7 regulates the abundance of LARS1 in a deubiquitinating enzyme activity‐dependent manner.

Then, we explored whether MOTS‐c affects USP7‐mediated deubiquitination of LARS1. It was observed that MOTS‐c treatment significantly reversed the endogenous deubiquitination of LARS1 caused by USP7 overexpression in A2780 and SKOV3 cells (Figure 4O). Similarly, MOTS‐c also largely counteracted the downregulation of LARS1 ubiquitination caused by USP7 overexpression in 293T cells (Figure S4B, Supporting Information). After that, we studied how this effect is achieved and found that MOTS‐c reduced the amount of LARS1‐bound USP7 but did not affect USP7 expression (Figure S4C,D, Supporting Information), suggesting that MOTS‐c may regulate LARS1 by blocking the binding of USP7 to LARS1, there may be a competition between MOTS‐c and USP7 for binding LARS1. The molecular docking model showed that MOTS‐c and USP7 both bound to the same amino acid residues D500 and D586 at LARS1, respectively, which meant that the binding interfaces of MOTS‐c and USP7 with LARS1 were mutually exclusive, indicating that MOTS‐c competed with USP7 for binding LARS1 (Figure S3E, Supporting Information). To further substantiate the point, different domains of LARS1 with WT USP7 (His‐USP7) were generated and over expressed in 239T cells. IP and IB assays showed that USP7 bound to the 256–605 amino acid region in LARS1 (Figure 4P), which was consistent with the region of MOTS‐c binding to LARS1. Based on our data, it is concluded that MOTS‐c competes with USP7 for binding LARS1. In conclusion, the above results indicate that MOTS‐c promotes the degradation of LARS1 by attenuating USP7‐mediated deubiquitination of LARS1.

### 2.5 LARS1 Is Increased in OC and its Upregulation Is Associated with Poor Patients’ Survival

**Figure 5:**
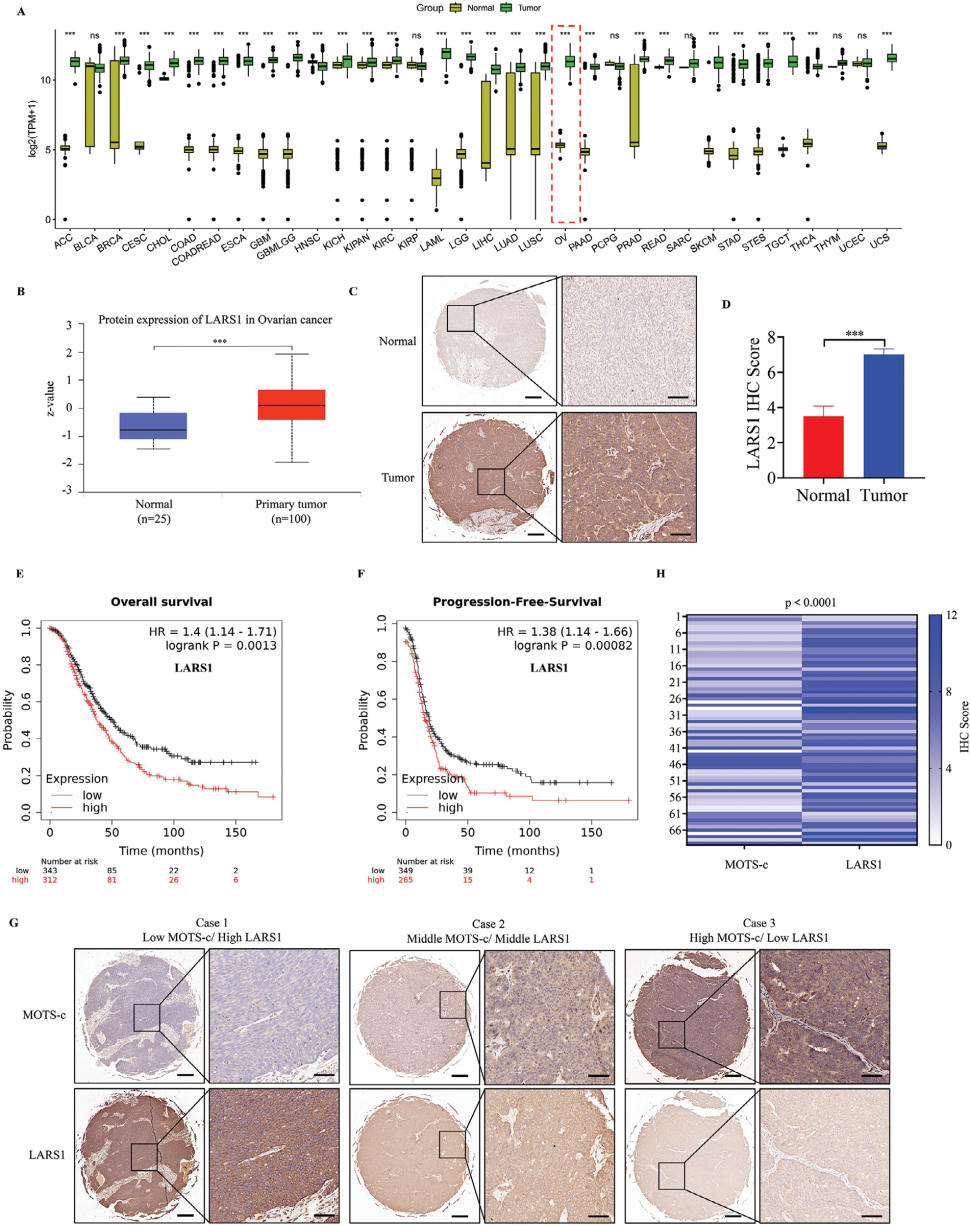
LARS1 is increased in OC and its upregulation is associated with poor patients’ survival. A) The mRNA levels of LARS1 in different tumors and normal tissues were analyzed by TCGA database. B) Protein levels of LARS1 in OC and normal tissues were analyzed by UALCAN platform. C,D) IHC analysis of LARS1 levels in OC and normal tissues. Scale bars, 100 and 20 µm. E,F) Using Kaplan–Meier plotter platform to analyze the (E) overall survival and (F) progression‐free survival of OC patients with high and low LARS expression. G) IHC staining of MOTS‐c and LARS1 in OC tissues, paraffin sections were derived from serial sections of the same wax block. H) Correlation analysis of MOTS‐c and LARS1 expression in OC tissues. Data are presented as the mean ± SEM, ^***^ *p* < 0.001.

A pan‐cancer analysis of LARS1 was performed using the TCGA database, which revealed that the mRNA levels of LARS1 were notably elevated in various tumors, including OC, compared to normal tissues (**Figure** **5**A). Then, the UALCAN platform was employed to conduct a proteomic analysis of OC tissues in the CPTAC database, and the results showed that LARS1 protein levels were greatly elevated in OC tissues as compared to normal tissues (Figure 5B). To confirm this, we analyzed the LARS1 expression in tissue microarrays with 80 samples (including ten normal ovarian tissues and 70 OC tissues) by IHC staining. The results showed that LARS1 expression was significantly higher in OC tissues than in normal controls (Figure 5C,D). Then, the association between LARS1 expression and the prognosis of OC patients was further analyzed by Kaplan–Meier plotter platform, and we found that high LARS1 expression was associated with shorter overall survival and progression‐free survival (Figure 5E,F, respectively). In addition, we also found that patients with low LARS1 expression had high MOTS‐c expression, whereas patients with high LARS1 expression had low MOTS‐c expression (Figure 5G). The expression levels of LARS1 were negatively correlated with MOTS‐c (Figure 5H) and positively correlated with USP7 (Figure S5A,B, Supporting Information), while there was no correlation between MOTS‐c and USP7 (Figure S5C, Supporting Information). These findings also affirmed our previous results, indicating that increased MOTS‐c expression may contribute to a downregulation of LARS1 in OC. Together, upregulated LARS1 may play a tumor‐promoting role during OC progression.

### 2.6 LARS1 Is a Cancer‐Promoting Factor in OC

**Figure 6:**
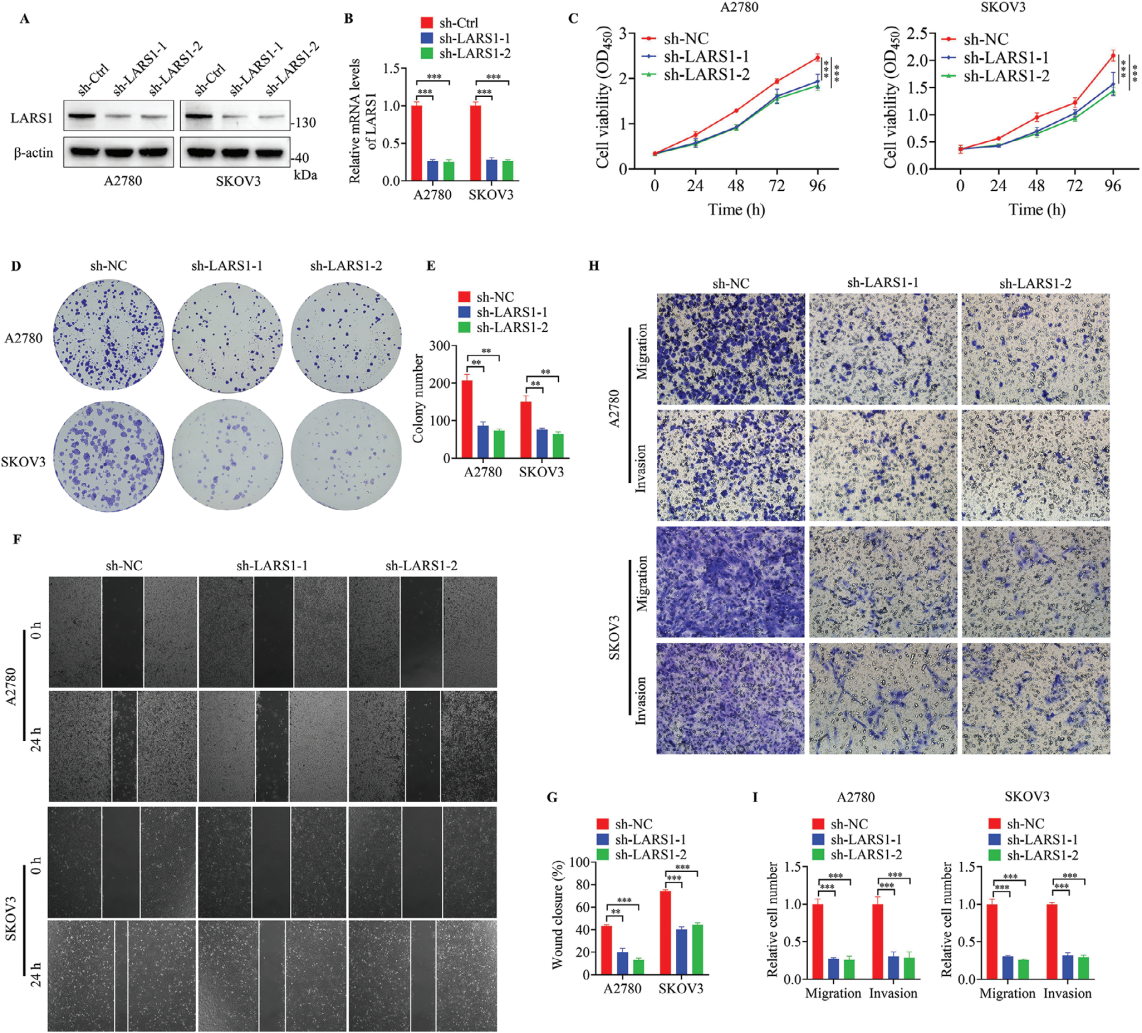
LARS1 is a cancer‐promoting factor in OC. A) Western blot and B) RT‐qPCR assays were conducted for analyzing the expression of LARS1 in OC cells transfected with LARS1 shRNA plasmids. C) CCK8, D,E) colony formation, F,G) wound healing, and H,I) transwell assays were conducted in OC cells with LARS1 knockdown. Data are presented as the mean ± SEM, ^**^ *p* < 0.01, ^***^ *p* < 0.001.

Although LARS1 has been shown to be a cancer‐promoting molecule in other tumors, its role in OC is unclear. To elucidate the function of LARS1 in OC, we generated a LARS1 shRNA plasmid and knocked down the expression of LARS1 in OC cells (**Figure** **6**A,B). Silencing of LARS1 markedly reduced the proliferative capacity of OC cells, as demonstrated by CCK8 and colony formation assays (Figure 6C–E). Meanwhile, the reduction of LARS1 levels also led to an obvious attenuation of the migration and invasion ability of OC cells compared to the negative control (Figure 6F–I). Collectively, these findings indicate that LARS1 is a cancer‐promoting factor in OC. However, exactly how LARS1 affects OC cells growth is still unclear. LARS1, as a known mTORC1 activator, and our previous data also confirmed that MOTS‐c can downregulate LARS1 protein levels and inhibit mTORC1 signaling in OC cells, suggesting that LARS1 is likely to influence the growth of OC cells through mTORC1 signaling. To confirm this, LARS1 was overexpressed in OC cells SKOV3 and treated with rapamycin (RAPA, a mTORC1 inhibitor), the results showed that RAPA significantly reversed the increased ability of proliferation, migration, and invasion caused by LARS1 overexpression (Figure S6A–G, Supporting Information). The above results suggest that LARS1 affects tumor progression by regulating mTORC1 signaling in OC.

### 2.7 Overexpression of LARS1 Reverses MOTS‐c‐Mediated Tumor Suppression in OC

**Figure 7:**
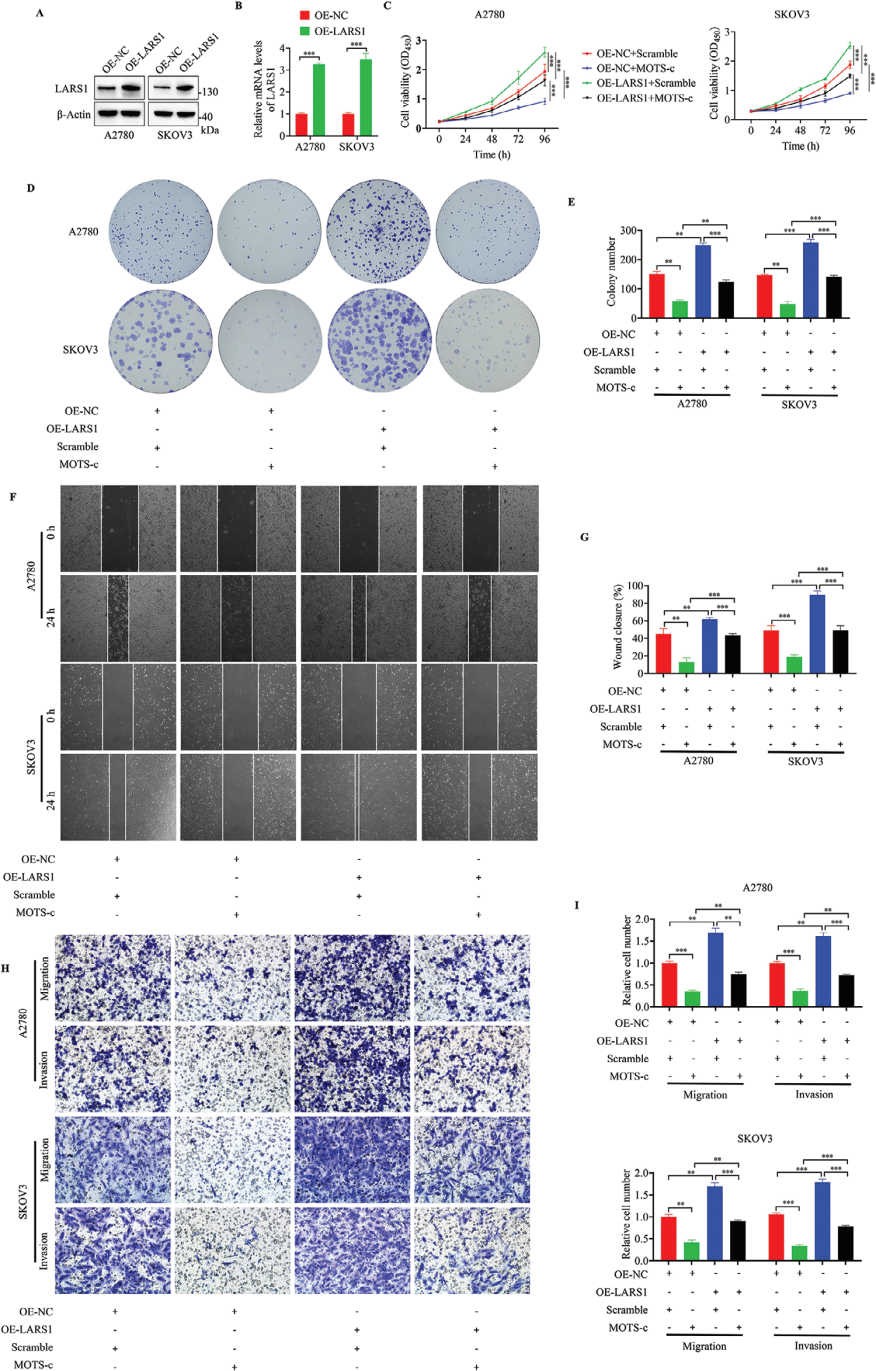
Overexpression of LARS1 reverses MOTS‐c‐mediated tumor suppression in OC. A) Western blot and (B) RT‐qPCR assays were conducted for analyzing the expression of LARS1 in OC cells transfected with LARS1 overexpression plasmid. C–I) OC cells transfected with LARS1 overexpressing plasmids or empty vector plasmids were treated with 30 µm MOTS‐c or Scramble, and subjected to (C) CCK8, (D, E) colony formation, (F, G) wound healing and (H, I) transwell assays, respectively. Data are presented as the mean ± SEM, ^**^ *p* < 0.01, ^***^ *p* < 0.001.

To further determine the relationship between the downregulation of LARS1 by MOTS‐c treatment and the proliferation, migration, and invasion of OC cells, we established A2780 and SKOV3 cells with stable overexpression of LARS1 (**Figure** **7**A,B). CCK8 and colony formation assays showed that overexpression of LARS1 significantly enhanced the proliferation of A2780 and SKOV3 cells, and attenuated or partially reversed the proliferation inhibition caused by MOTS‐c treatment (Figure 7C–E). Similarly, the results of wound healing and transwell assays showed that increased levels of LARS1 notably enhanced the migratory and invasive capabilities of OC cells, leading to the counteraction of MOTS‐c effects (Figure 7F–I). These findings suggest that MOTS‐c inhibits the progression of OC by targeting LARS1.

### 2.8 MOTS‐c Displayed a Marked Anti‐Tumor Effect on OC Growth Without Systemic Toxicity In Vivo

**Figure 8:**
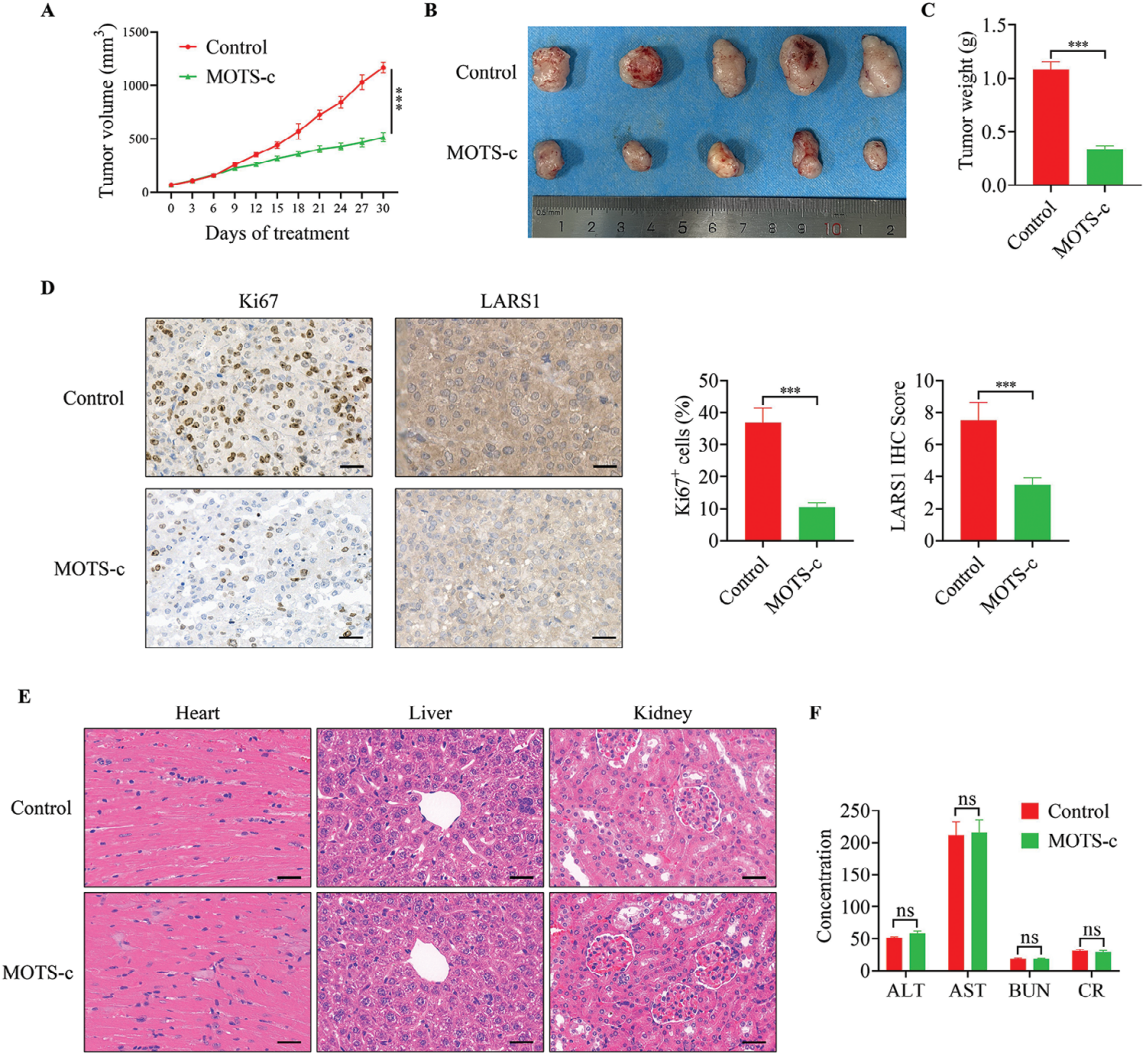
MOTS‐c displayed a marked anti‐tumor effect on OC growth without systemic toxicity in vivo. A) Growth volume curves of A2780 cell xenografts in Control and MOTS‐c treated groups. B,C) After 30 days of treatment, nude mice were executed, and tumors were excised, photographed, and weighed. D) IHC staining for Ki67 and LARS1 in subcutaneous xenograft tumors. E) H&E staining of heart, liver, and kidney tissues. F) Detection of serum alanine transaminase (ALT), aspartate aminotransferase (AST), blood urea nitrogen (BUN), and creatinine (CR) levels in two groups. Data are presented as the mean ± SEM, ^***^ *p* < 0.001.

To evaluate the anti‐tumor effect of MOTS‐c in vivo, we constructed a xenograft model of female BALB/c‐nude mice using A2780 cells. The results showed that MOTS‐c treatment significantly inhibited the growth of OC cells and reduced the tumor volume and weight (**Figure** **8**A–C). IHC analysis showed that MOTS‐c treatment inhibited the proliferation of tumor cells, and the Ki67 positive cell ratio was significantly reduced compared to the control group. Meanwhile, the expression of LARS1 was notably reduced after MOTS‐c treatment (Figure 8D). In addition, to evaluate whether MOTS‐c has drug toxicity in vivo, we collected the hearts, livers, and kidneys for H&E staining, and collected serum samples for biochemical testing from two groups of mice. No evidence of obvious toxicity was found after MOTS‐c treatment in the analysis of pathological sections and biochemical results (Figure 8E, F, respectively). Collectively, the above results demonstrate that MOTS‐c could inhibit the growth of OC cells without toxicity in vivo.

## 3 Discussion

In the present study, we found for the first time that MOTS‐c serves as an anti‐tumor peptide to inhibit OC progression. MOTS‐c levels were significantly reduced in both serum and tumor tissues from OC patients, and low MOTS‐c expression in OC tissues was associated with a poor patients’ prognosis. In vitro, exogenous MOTS‐c could dose‐dependently inhibit the proliferation, migration and invasion of OC cells, and induce cell cycle arrest and apoptosis. Mechanistically, we identified LARS1 as a potential binding target of MOTS‐c through a biotin pull‐down assay combined with MS analysis, and MOTS‐c could promote the degradation of LARS1 through the ubiquitin‐proteasome pathway. Further studies revealed that USP7 functioned as a deubiquitinase and stabilizer of LARS1, while MOTS‐c attenuated USP7‐mediated LARS1 deubiquitination by competing with USP7 for binding LARS1, leading to its degradation. In addition, we found that LARS1 has significantly high expression and important oncogenic function in OC. More importantly, MOTS‐c displayed an obvious anti‐tumor effect on OC growth without systemic toxicity in vivo. In conclusion, our study elucidated the function and mechanism of MOTS‐c in human cancers for the first time, providing a new possibility for the treatment of OC.

MOTS‐c is gaining increasing attention as an endogenous small molecule peptide of mitochondrial origin and is gradually emerging as a possible link to tumor development. It has been found that MOTS‐c directly blocked purine synthesis from scratch by inhibiting the folate‐methionine cycle, thereby increasing AICAR (an AMPK activator) levels, activating AMPK, increasing cellular glucose uptake, accelerating ATP production, and leading to the inhibition of mitochondrial respiration, an action that shares many similarities with the antifolate drug methotrexate, which was initially developed for the treatment of malignant tumors.^[^ ^7^, ^15^ ^]^ Meanwhile, besides inhibiting the proliferation of immortalized HEK293 cells, MOTS‐c also prevented diet‐induced obesity and insulin resistance in mice as well as age‐related insulin resistance.^[^ ^7b^ ^]^ Recently, studies have revealed that MOTS‐c can translocate into the nucleus and regulate nuclear gene expression.^[^ ^7^, ^16^ ^]^ In the resting state, endogenous MOTS‐c was mainly distributed outside the nucleus. When cells were stimulated by metabolic stress, cytoplasmic MOTS‐c could rapidly translocate into the nucleus through an AMPK‐dependent process. Once inside, it would bind to the stress‐responsive transcription factor NRF2 and its associated genes to boost the cells’ ability to withstand metabolic stress and maintain cellular homeostasis.^[^ ^7a^ ^]^ However, in this study, NRF2 was not identified in our mass spectrometry results, suggesting that MOTS‐c may not bind NRF2 in OC cells. Coincidentally, a recent study showed that MOTS‐c also did not bind NRF2 in hepatocytes.^[^ ^17^ ^]^ These phenomena suggest that MOTS‐c may bind to different molecules to perform various functions in different cells. In addition, tumors are a disease associated with aging, and circulating MOTS‐c levels have been found to decrease with age.^[^ ^16^ ^]^ Moreover, exogenous MOTS‐c reversed age‐related senescence and prolonged survival time in aged mice, an effect that may be related to the entrance of exogenous MOTS‐c into the nucleus to activate the transcription factor HSF1 and thus heat shock protein expression.^[^ ^16^ ^]^ Furthermore, an increasing amount of research indicates a notable decrease in MOTS‐c levels in cancer patients. Serum MOTS‐c levels were markedly downregulated in patients with prostate cancer and hepatocellular carcinoma compared to healthy individuals.^[^ ^8^, ^10^ ^]^ Additionally, MOTS‐c has been linked to the likelihood of developing prostate and breast cancer, showing variations based on race.^[^ ^10^ ^]^ There was a notable rise in MOTS‐c levels following radiotherapy in individuals with lung cancer.^[^ ^9^ ^]^ A recent study discovered that MOTS‐c levels were decreased in hepatitis B virus‐associated hepatocellular carcinoma tissues, and exogenous MOTS‐c could protect liver function by promoting mitochondrial fusion remodeling and inhibiting hepatitis B virus DNA replication.^[^ ^8^ ^]^ Similarly, we compared the differences in serum MOTS‐c levels between healthy women and OC patients of the same age group and found that circulating MOTS‐c levels were markedly downregulated in OC patients, and the expression of MOTS‐c was also significantly lower in OC tissues. Low levels of MOTS‐c were associated with shorter overall and disease‐free survival. These results are a strong signal that MOTS‐c may be an important molecule influencing tumor progression. Unfortunately, none of the above studies have determined whether there is a relationship between circulating and tissue MOTS‐c, nor did they investigate the relationship between circulating MOTS‐c levels and tumor stage or prognosis. We know that MOTS‐c is a mitochondrial polypeptide produced in the cytoplasm and can be secreted into the circulation to exert mitochondrial hormone‐like effects. Mitochondrial dysfunction is characteristic of tumors. Hence, it is worthwhile to clarify in the future whether the reduction in circulating MOTS‐c levels in tumor patients is due to reduced secretion into the bloodstream due to low expression of MOTS‐c in tumor cells, or whether tumor patients as a whole have reduced levels of MOTS‐c expression, which opens up opportunity using circulating MOTS‐c as a non‐invasive means of predicting tumorigenesis or determining prognosis, which needs to be explored in larger clinical studies. In addition, in this study we failed to elucidate the reason for the downregulation of MOTS‐c in OC, and the mechanism by which MOTS‐c is regulated is still unclear, which is vitally important for the study of the mechanism of the role of MOTS‐c in tumors. So as a next step, researchers should not only focus on the function of MOTS‐c itself, but should also explore the pathways that regulate MOTS‐c expression.

Leucyl‐tRNA synthetase 1 (LARS1), a member of the class I aminoacyl‐tRNA synthetase family is the enzyme responsible for attaching leucine to its corresponding tRNA. LARS1 primarily functions by utilizing ATP to create Leu‐AMP and activate leucine, facilitating its bonding with tRNA. Leucine and tRNA catalyze the formation of leucyl‐tRNA, which is used for ribosome translation and provides precursor material for the next step of protein synthesis.^[^ ^12a^ ^]^ Research has demonstrated that LARS1 possesses GTPase activity and translocates to the lysosome when leucine is present, to convert inactive RagD GTP to active RagD GDP, ultimately activating mTORC1.^[^ ^18^ ^]^ The process of mTORC1 activation contributes to tumorigenesis.^[^ ^19^ ^]^ Multiple studies have indicated that LARS1 could serve as a promising candidate for cancer‐fighting medications.^[^ ^13^, ^20^ ^]^ Silencing of LARS1 significantly inhibited the growth of osteosarcoma cells and lung cancer.^[^ ^13^ ^]^ Additionally, the LARS1 inhibitor BC‐LI‐0186 demonstrated anti‐tumor properties in non‐small cell lung cancer, especially when used in conjunction with the MEK inhibitor trametinib.^[^ ^21^ ^]^ In contrast, the knockout of a single allele of the LARS1 gene in MMTV‐PyMT mice, a breast cancer model, showed that LARS deletion promoted tumor growth and increased tumor load in mice.^[^ ^22^ ^]^ In addition, LARS1 expression was regulated by PGC‐1α in colorectal cancer cells and promoted the proliferation, migration, and invasion of colorectal cancer cells by regulating the expression of cyclin D1, c‐Myc, and waveform protein.^[^ ^20b^ ^]^ A recent study found that high expression of LARS1 was strongly associated with poor prognosis and stage of hepatocellular carcinoma.^[^ ^23^ ^]^ In this study, we found that LARS1 is a pro‐carcinogenic molecule in OC, and the knockdown of LARS1 inhibited the malignant progression of OC cells. We identified a possible interaction between MOTS‐c and LARS1 by biotin‐pulldown and MS analysis, which was further confirmed by Co‐IP, Western blot, and IF. We then found that MOTS‐c treatment reduced LARS1 protein levels, while not impacting its mRNA levels, suggesting that the regulation was achieved post‐transcriptionally. CHX experiments confirmed that MOTS‐c accelerated the degradation of LARS1 protein, and MG132 and CQ experiments confirmed that this effect was dependent on the proteasome pathway. Finally, by IP and Western blot, we confirmed that MOTS‐c caused LARS1 degradation through the ubiquitin‐proteasome pathway. Furthermore, it's worth mentioning that post‐translational modifications of aminoacyl‐tRNA synthetases may affect their own catalytic activity,^[^ ^24^ ^]^ thus the regulation of LARS1 by MOTS‐c may not only be limited to ubiquitination, which down‐regulated mTORC1 signaling by affecting the stability of the LARS1 protein, thereby affecting ovarian cancer progression (the non‐classical pathway); but it may also act by affecting the catalytic activity of LARS1 (the classical pathway). In addition, although our data showed MOTS‐c regulated post‐translational modifications of the LARS1 protein and did not affect its mRNA levels, we also found that the mRNA levels of LARS1 varied in ovarian cancers, suggesting that the regulation of LARS1 may not only occur at the post‐translational level, but also at a transcriptional or post‐transcriptional level. Just as the well‐known gene PD‐L1 (CD274), which is subject to both transcriptional and post‐transcriptional regulation, as well as post‐translational regulation simultaneously in the same tumor.^[^ ^25^ ^]^ LARS1 is an important metabolic regulatory molecule, and the current research on how its expression is regulated is very limited. We believe that more data will be added in the future, and we are looking forward to more answers to LARS1 regulation.

Ubiquitination is a reversible type of post‐translational modification of proteins. Ubiquitin‐activating enzymes (E1s) first attach to ubiquitin to activate it, then pass the activated ubiquitin to ubiquitin‐conjugating enzymes (E2s), and finally, ubiquitin ligases (E3s) move the ubiquitin from the E2 to the target, with E3 ligases being crucial in the entire ubiquitination process.^[^ ^26^ ^]^ In contrast, deubiquitinating enzymes (DUB) catalyze the removal of ubiquitin from deubiquitinated proteins, thereby reversing the ubiquitination process.^[^ ^27^ ^]^ Dysregulation of ubiquitination and ubiquitination leads to a variety of diseases, including tumors.^[^ ^28^ ^]^ In this study, we found that MOTS‐c promoted the ubiquitination and degradation of LARS1. However, since MOTS‐c was not an E3 ligase or deubiquitinating enzyme, we speculated that there may be an E3 ligase or a deubiquitinating enzyme that played a “middleman” role between MOTS‐c and LARS1. By IP and MS, we found that the deubiquitinating enzyme USP7 could bind to both MOTS‐c and LARS1, which was further confirmed by IB and IF assays. Knockdown of USP7 promoted the degradation of LARS1, whereas overexpression of USP7 stabilized LARS1, confirming that USP7 is a LARS1‐specific deubiquitinating enzyme, and MOTS‐c reverses the deubiquitination caused by USP7 overexpression. Next, we immunoprecipitated LARS1 protein after MG132 treatment and examined the effect of MOTS‐c on the ability of LARS1 to bind USP7. The results confirmed that MOTS‐c significantly reduced the binding of USP7 to LARS1. By constructing mutants with different structural domains of LARS1, MOTS‐c, and USP7 were found to bind the same region on LARS1, indicating MOTS‐c competes with USP7 for binding LARS1, which was also supported by the molecular docking model. Similarly, some studies also reported that USP7 can compete with other proteins for binding substrates.^[^ ^29^ ^]^ Based on our data, we concluded that MOTS‐c competed with USP7 for binding LARS1, thereby impairing the deubiquitination of LARS1 and ultimately leading to LARS1 degradation.

## 4 Conclusion

**Figure 9:**
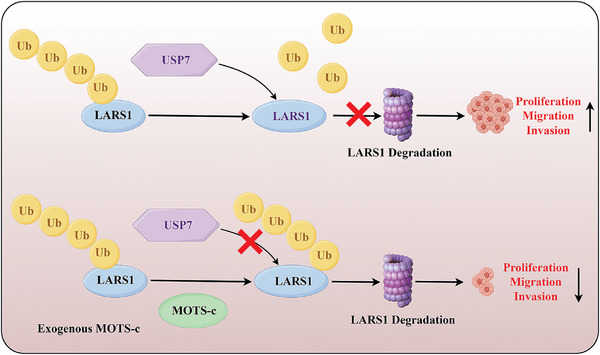
Graphical summary of the proposed mechanism.

In summary, we have confirmed that MOTS‐c expression is reduced in OC and low MOTS‐c expression is associated with poor prognosis. Exogenous MOTS‐c can inhibit OC cell growth in vitro and in vivo. Mechanistically, MOTS‐c exerted anti‐cancer effects by attenuating USP7‐mediated deubiquitination of LARS1 and promoting LARS1 degradation (**Figure** **9**). Our study revealed for the first time that MOTS‐c serves as an antitumor peptide to inhibit OC progression, and thus could be a potential target for future OC diagnosis and treatment.

## 5 Experimental Section

### Clinical Specimens

In this study, 80 serum samples (40 from healthy women and 40 from OC patients) and 247 OC specimens were obtained from Xijing Hospital, the First Affiliated Hospital of Air Force Medical University. These samples were approved for use by the Ethics Committee of Xijing Hospital, the First Affiliated Hospital of Air Force Medical University, and written informed consent was signed by all participants. In addition, 80 cases of OC tissue chips were purchased from Bioaitech Co., Ltd.

### Enzyme‐Linked Immunosorbent Assay (ELISA)

In this study, a commercial ELISA kit (Cloud‐Clone Corp, China, #CEX132Hu) was used for detecting the MOTS‐c levels in serum from ovarian cancer patients. Operations were carried out according to kit instructions.

### Immunohistochemistry (IHC)

For IHC, in brief, after dehydration, antigen repair, inhibition of endogenous peroxidase, and serum blocking treatment, paraffin sections were incubated with primary antibodies against MOTS‐c (#MOTSC‐101AP, 1:50, FabGennix, USA), LARS1 (#21146‐1‐AP, 1:200, Proteintech, China), USP7 (#66514‐1‐lg, 1:200, Proteintech, China) and Ki67 (#27309‐1‐AP, 1:10 000, Proteintech, China) overnight at 4 °C. Then, after incubating with an HRP‐conjugated anti‐rabbit or anti‐mouse secondary antibody, the specimens were stained with diaminobenzidine (DAB) and hematoxylin. All sections were scored by two pathologists according to the immunoreactive scoring (IRS) system.

### Western Blot

For tissue proteins, fresh OC tissue was detached and frozen in liquid nitrogen. Then, RIPA lysis solution containing protease inhibitors was added for tissue grinding, followed by centrifugation to extract the supernatant. Electrophoresis was performed using a commercially available polyacrylamide electrophoresis gel at a concentration of 4–20% (ACE Biotechnology, China, #ET15420Gel), and then transferred the protein to a PVDF membrane (Immobilon, USA, #ISEQ00010). After sealing with 5% skimmed milk for 1 h, the membrane was incubated with primary antibody at 4 overnight. After that, the membrane was incubated with a secondary antibody at room temperature for 1 h, washed with TBST for 10 min thrice, and captured the Western blot images using a chemiluminescence imaging system. The extraction of cell proteins and the process of immunoblotting analysis are the same as the previous protocol.^[^ ^30^ ^]^ The incubated antibodies include MOTS‐c (#MOTSC‐101AP, 1:500, FabGennix, USA), β‐actin (#81115‐1‐RR, 1:10 000, Proteintech, China), Bcl‐2 (#381 702, 1:1000, ZEN‐BIOSCIENCE, China), Bax (#380 709, 1:1000, ZEN‐BIOSCIENCE, China), Cleaved‐PARP (#380 374, 1:1000, ZEN‐BIOSCIENCE, China), Cleaved‐Caspase3 (#341 034, 1:1000, ZEN‐BIOSCIENCE, China), LARS1 (#21146‐1‐AP, 1:1000, Proteintech, China), Ubiquitin (#10201‐1‐AP, 1:1000, Proteintech, China), Flag (#66008‐4‐lg, 1:5000, Proteintech, China), GFP (#50430‐2‐AP, 1:1000, Proteintech, China), p‐mTOR (#5536, 1:1000, CST, USA), mTOR (#2983, 1:1000, CST, USA), p‐S6K (#9205, 1:1000, CST, USA), S6K (#2708, 1:1000, CST, USA), p‐4EBP1 (#2855, 1:1000, CST, USA), 4EBP1 (#9644, 1:1000, CST, USA), USP7 (#66514‐1‐lg, 1:5000, Proteintech, China) and His (#66005‐1‐lg, 1:5000, Proteintech, China).

### Cell Lines and Cell Culture

The human OC cell lines A2780 and SKOV3 were cultured in RPMI 1640 (Gibco, China). The 293T cell was maintained in DMEM (Gibco, China). All culture media are supplemented with 10% fecal body serum (FBS), 100 U mL^−1^ penicillin, and 0.1 mg mL^−1^ streptomycin. Humidified air containing 5% CO_2_ was utilized for growing the cells at 37 °C. The cell lines used in this study were purchased from the American Type Culture Collection (ATCC, China) and were authenticated by short tandem repeat (STR) to confirm their identity and purity.

### Cellular Uptake of Peptides

The OC cells were incubated with FITC‐labeled MOTS‐c (10 µm) for 3 h. Then, under dark conditions, fix the cells with 4% paraformaldehyde, stain the nuclei with DAPI, and finally observe and take photos under a fluorescence microscope.

### Cell Counting Kit‐8 Assay (CCK8)

The OC cells were seeded into 96‐well plates at the density of 2 × 10^3^ cells per well, and corresponding concentrations of peptides were added into the medium according to the set groups. Each group had six multiple wells and was cultured for 0, 24, 48, 72, and 96 h. A total of 10 µL Cell Counting Kit‐8 (CCK8) was added to each well and incubated in the dark for 1 h and was measured at the optical density (OD) at 450 nm.

### Colony Formation

The OC cells were seeded in a 6‐well plate at a density of 500 cells per well. The cell culture medium was changed every three days. After 12 days, the colonies were fixed with 4% paraformaldehyde for 15 min and stained with crystal violet for 15 min. Colonies in each well were photoed and then counted by Image J.

### Wound Healing

The OC cells were seeded in a 6‐well plate and cultured in medium supplemented with 10% FBS for ≈24 h to 90% confluence. Then, a scratch was made in a straight line along the diameter of the well using a 1000 µL pipette tip, and the medium was replaced with serum‐free medium. Images of the wound healing process were captured at 0 h and 24 h using an inverted microscope and the wound healing area was measured using Photoshop.

### Transwell Migration and Invasion Assays

Transwell chambers (#TCS‐003‐024, BIOFIL, China) equipped with 8.0 µm pore inserts with or without a matrigel coating (#082704, ABW, China) were employed. Briefly, the OC cells were resuspended in serum‐free medium and seeded in the upper cell chamber at a density of 2 × 10^4^ cells per well, while medium supplemented with 10% FBS was added to the lower cell chamber. After 24 or 48 h of incubation, the cells in the chambers were fixed with 4% paraformaldehyde for 20 min and stained with crystal violet for 20 min, and take photos of transwell chambers under an inverted microscope and count cells using Image J.

### Flow Cytometry Analysis

Cell cycle and cell apoptosis assays in OC cells were carried out according to the manufacturer's instructions using the Cell Cycle and Apoptosis Analysis Kit (#550825, #556547, BD Pharmingen, USA) after the indicated treatments. The data were analyzed by ModFit 3.0 and EXPO32 ADC Analysis.

### Peptide Pull Down and Mass Spectrometry Analysis

Biotin‐labeled MOTS‐c (Biotin‐MOTS‐c, 200 µg) and biotin‐labeled scramble peptide (Biotin‐Scramble, 200 µg) were separately incubated with 30 uL streptavidin Dynabeads (#11205D, Invitrogen, USA) at 4 °C for 12 h, then the beads were washed 6 times with RIPA lysis solution containing protease and phosphatase inhibitors. Collected the protein of A2780 cells, incubated 2 mg protein and the beads at 4 °C for another 12 h, then washed the beads as the above. Finally, eluted beads using 30 uL 1×loading buffer at 95 °C for 10 min. The eluted complexes were detected by Coomassie blue staining and mass spectrometry. Mass spectrometry was performed by BGI (Shenzhen, China).

### Co‐Immunoprecipitation (Co‐IP)

Firstly, antibodies (anti‐LARS1, anti‐USP7, anti‐Flag, 10 µg) or IgG were incubated with 50 uL rProtein A/G magnetic beads for 12 h at 4 °C. Cells were lysed using RIPA lysis buffer containing protease inhibitors and centrifuged to obtain protein supernatant. After washing with RIPA lysis, the cell lysate was incubated with beads for 12 h at 4 °C, and the protein was extracted from the beads by boiling at 95 °C for 10 min. The samples were then detected by Western blot analysis.

### Cell Transfection

For stable transfection, the LARS1 shRNA plasmids, Flag‐LARS1 plasmids, HA‐Ub plasmids (WT, K6, K11, K27, K29, K33, K48, K63, K6R, K11R, K27R, K29R, K33R, K48R, K63R), site‐mutant plasmids of LARS1 (K243A, K329A, K341A, G338A, I499A, D500A, D586A, D500A&D586A), LARS1 truncated plasmids (1‐255, 256–1176, 606–1176, 764–1176, 893–1176, and 1064–1176), GFP‐MOTS‐c plasmids, His‐USP7 plasmids and His‐USP7‐C223S plasmids as well as each control plasmid were purchased from GeneChem Corporation (Shanghai, China). LARS1 target sequences included: shRNA‐1: 5ʹ‐GCTGTGCTTATGGAGAATATA‐3ʹ, shRNA‐2: 5ʹ‐CCTCACTTTGACCCAAGCTAT‐3ʹ. For transient transfection, USP7 siRNAs were designed and synthesized by Sangon Bioengineering (Shanghai) Co., Ltd., and sequences of siUSP7‐1 was 5ʹ‐UAGCUUCAUGCAACAUGAUTT‐3ʹ and siUSP7‐2 was 5ʹ‐CUGGUUCAUAGUGGAGAUATT‐3ʹ. Transfections were carried out using the lipofectamine 3000 reagent (#L3000015, Invitrogen, USA) according to the standard protocols. The transfection efficiency was determined by RT‐qPCR and Western blot analyses.

### RNA Extraction and RT‐qPCR

RNA extraction was obtained using the kits according to the instruction (#MF036‐01, Mei5bio, China), and the RNA was reversed to cDNA by using the kits (#AG11706, AGBIO, China). Then cDNA was amplified using specific primers. Primer sequences are listed as follows:

LARS1 (forward primer, 5ʹ‐GAAATAGAGCTGTATGGTTGCCC‐3ʹ; reverse primer, 5ʹ‐TCAAGCCAATGTTCTGCTTCA‐3ʹ), USP7 (forward primer, 5ʹ‐GGAAGCGGGAGATACAGATGA‐3ʹ; reverse primer, 5ʹ‐AAGGACCGACTCACTCAGTCT‐3ʹ), and ACTB/Actin Beta (forward primer, 5ʹ‐GCTGTGCTATCCCTGTACGC‐3ʹ; reverse primer, 5ʹ‐TGCCTCAGGGCAGCGGAACC‐3ʹ). Using Actin Beta as an internal reference, relative changes in gene expression were normalized against Actin Beta.

### Animal Models

BALB/c nude female mice (4–5 weeks old) were purchased from Beijing Vital River Laboratory Animal Technology Co., Ltd., and all animal experiments were in accordance with guidelines from the Institutional Animal Care and Use Committee of the Air Force Medical University. Mice had 5 × 10^6^ A2780 cells in 100 uL suspension (PBS: Matrigel = 1:1)injected subcutaneously into their left fanks. Seven days after inoculation, tumor size was measured by a caliper every 3 days. The mice were randomly separated into the following two groups (n = 5 in each group): Control group (injected with PBS) and MOTS‐c group (injected with 20 mg kg d^−1^ MOTS‐c). Intraperitoneal injection on time every day for 30 consecutive days. All mice were euthanized 30 days after initial treatment, serum was collected from each mouse for biochemical tests; heart, liver, and kidney sections were fixed with 4% paraformaldehyde for H&E Staining; the tumors were harvested and weighted using double‐blinded evaluation. Partial tumor tissues were fixed with 4% paraformaldehyde and embedded in paraffin for IHC staining.

### Statistics

All data are expressed as the mean ± SEM. The differences between the two groups were analyzed using a two‐tailed Student's t‐test performed on GraphPad Prism 9. *P*‐values < 0.05 were considered significant. For every figure, statistical tests are justified as appropriate.

## Acknowledegments

This work was funded by grants from the National Natural Science Foundation of China (Grant No. 82172993), Shaanxi Provincial Natural Science Basic Research Program. (Grant No. 2022JQ‐977), Shaanxi Provincial Innovation Platform (Grant No. 2023PT‐07), and Shaanxi Provincial Key Industry Innovation Chain (Grant No. 2024SF‐ZDCYL‐01‐17).

## Conflict of Interest

The authors declare no conflict of interest.

## Author Contributions

Y.Y., Y.L., B.M., and C.R. contributed equally to this work. Y.D.Y. conceived research ideas, designed and performed experiments, analyzed data, and wrote the original manuscript. Y.J.L. performed data collection and manuscript revisions. B.Y.M. and C.L.R. analyzed data. S.H.Z., J.L., and Y.G. collected clinical samples. H.Y. provided the funding and approved the final version of the manuscript. J.B.L. reviewed and edited the manuscript, and helped in completing experiments.

## Supporting information



Supporting Information

## Data Availability

The data that support the findings of this study are available from the corresponding author upon reasonable request.
