# Supplementary material for: Mitochondrial‐Derived Peptide MOTS‐c Suppresses Ovarian Cancer Progression by Attenuating USP7‐Mediated LARS1 Deubiquitination
Source: Adv Sci (Weinh). 2024 Sep 25;11(43):2405620. doi: 10.1002/advs.202405620 (PMC11578304; doi:10.1002/advs.202405620)
Supplement: Supplementary file 1 — Supporting Information [file ADVS-11-2405620-s001.docx]

**Supporting Information**

**Table S1.** Correlation between the expression levels of MOTS-c and clinicopathologic features of 247 ovarian cancer patients.

| Variables | MOTS-c expression | | NO. of Patients | *p* value |
| --- | --- | --- | --- | --- |
|  | low | high |  |  |
| All | 150 | 97 | 247 |  |
| Age (year) |  |  |  |  |
| ≤ 52 | 68 | 56 | 124 | 0.057 |
| ＞52 | 82 | 41 | 123 |  |
| FIGO stage |  |  |  |  |
| I-II | 28 | 34 | 62 | 0.004 |
| III-IV | 122 | 63 | 185 |  |
| Tumor size (cm) |  |  |  |  |
| ≤ 5 | 56 | 42 | 98 | 0.349 |
| ＞5 | 94 | 55 | 149 |  |
| Lymphatic invasion |  |  |  |  |
| No | 89 | 73 | 162 | 0.010 |
| Yes | 61 | 24 | 85 |  |


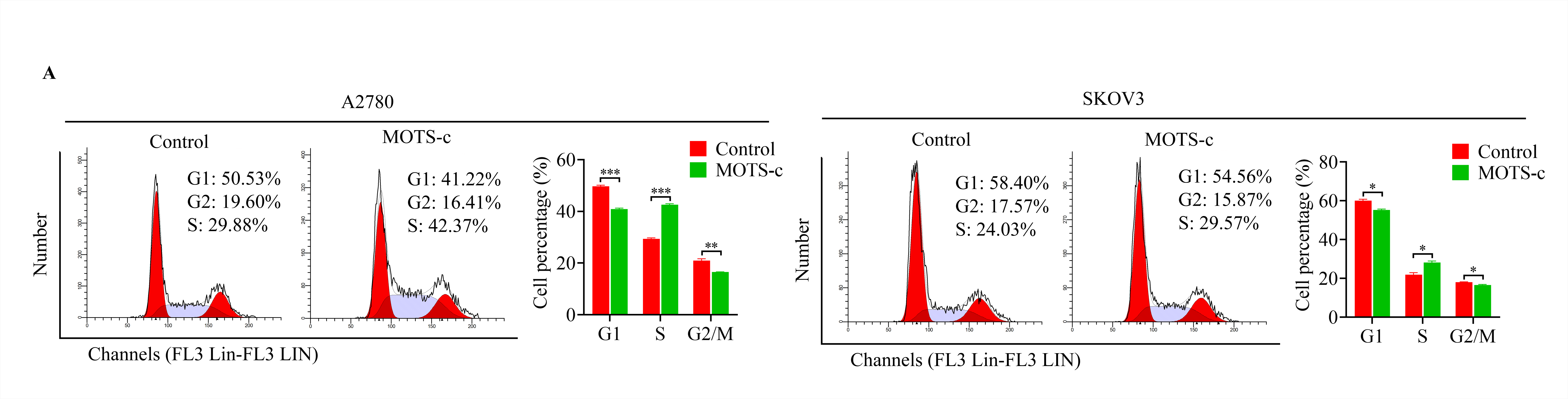


**Figure S1.** (A) Flow cytometry assay was conducted to analyze the effects of MOTS-c (30 μM) treatment on the cell cycle of A2780 and SKOV3 cells. **p* < 0.05, ***p* < 0.01, ****p* < 0.001.


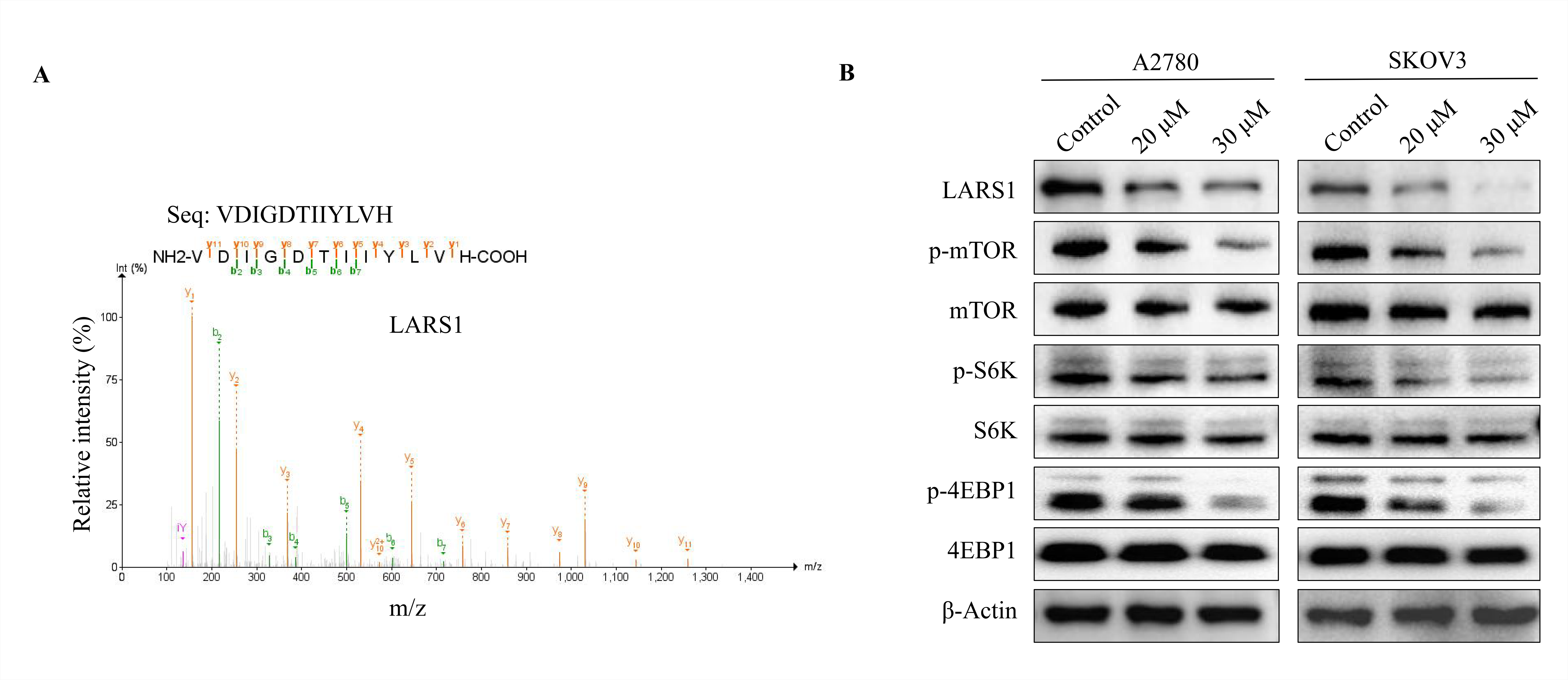


**Figure S2.** (A) A mass spectrogram of LARS1 protein. (B) Western blot assay was conducted to analyze the expression levels of LARS1, p-mTOR, mTOR, p-S6K, S6K, p-4EBP1, and 4EBP1 in LARS1/mTORC1 signaling pathway in A2780 and SKOV3 cells treated with 20 μM and 30 μM MOTS-c.

**
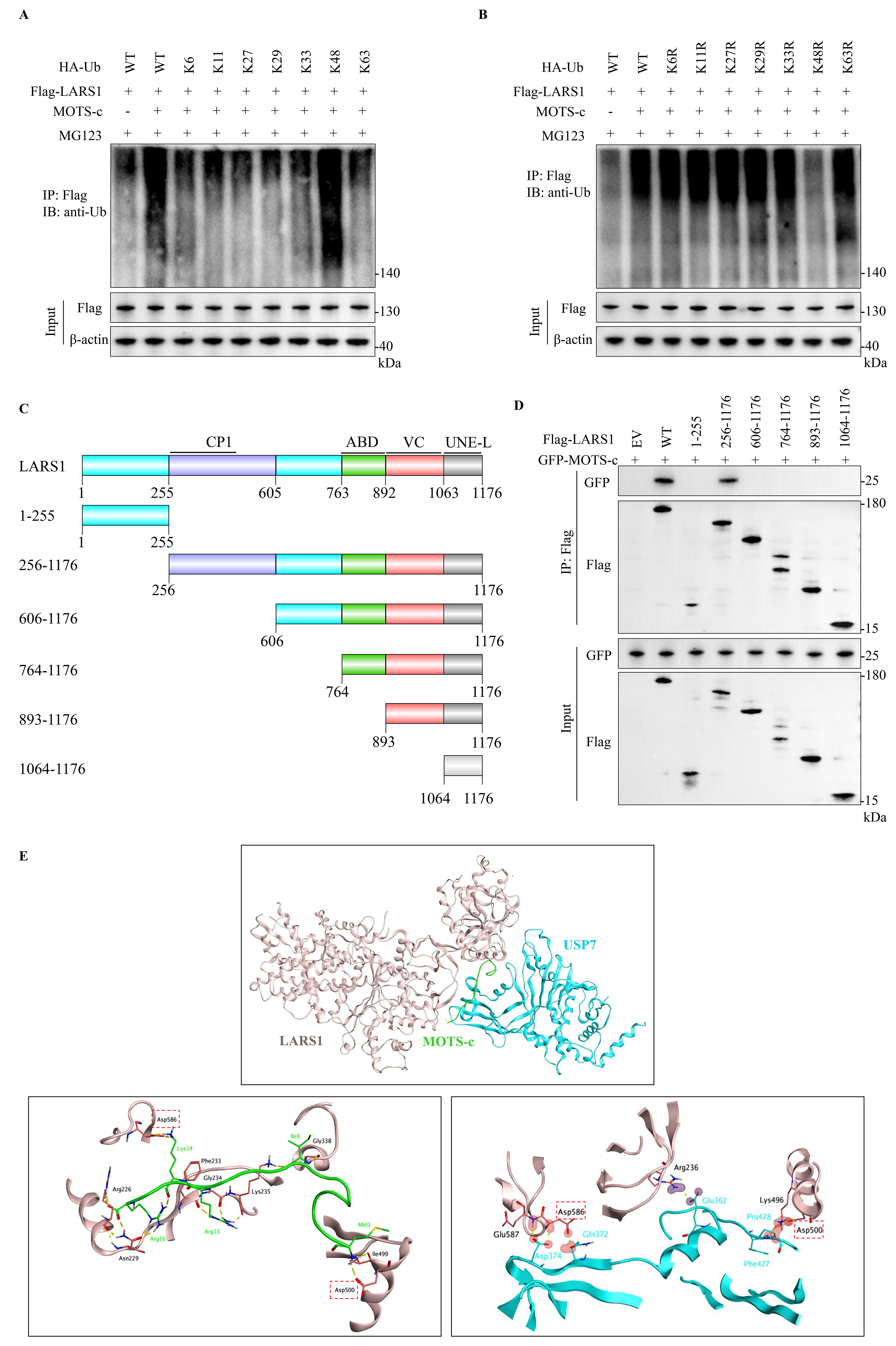
**

**Figure S3.** (A, B) IP and IB analysis of the effect of MOTS-c on specific ubiquitination types of LARS1. (A) Wild type ubiquitin (HA-Ub (WT)) and single lysine residue-only ubiquitin (K6-, K11-, K27-, K29-, K33-, K48-, and K63-ubiquitin) were overexpressed in 293 T cells. Cells were first treated with MOTS-c, 20 μM MG132 was then added 8 hours before harvesting. (B) HA-Ub (WT) and the single lysine residue mutant ubiquitin (K to R) were overexpressed in 293 T cells. Cells were first treated with MOTS-c, 20 μM MG132 was then added 8 hours before harvesting. (C) Schematic representation of various LARS1 truncations. (D) IP and IB analysis of the binding of MOTS-c to different structural domains of LARS1. (E) Prediction of the molecular docking model for LARS1, MOTS-c and USP7. The binding sites between MOTS-c and LARS1, and between USP7 and LARS1 were predicted by MOE software.


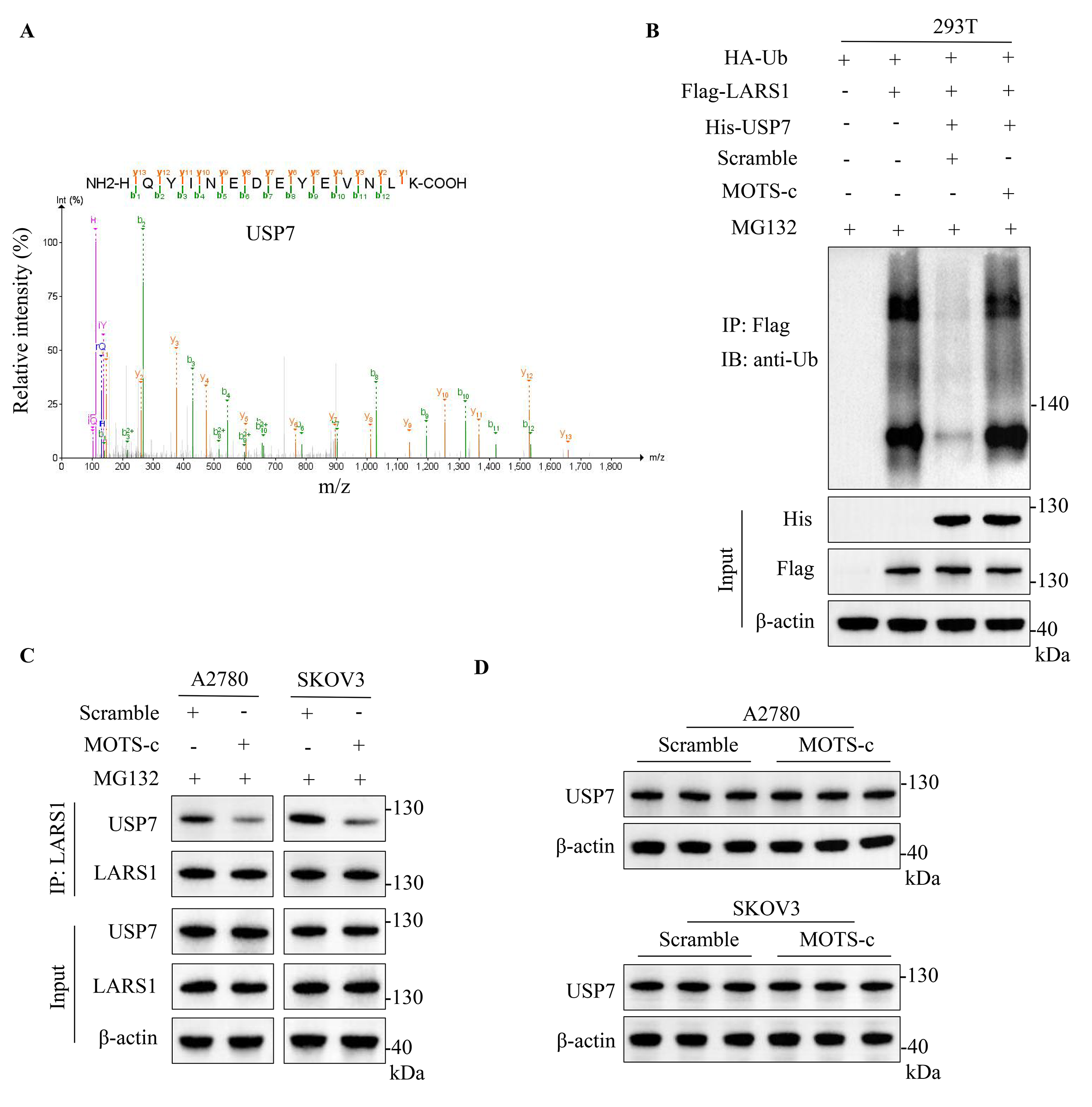


**Figure S4.** (A) A mass spectrogram of USP7 protein. (B) IP and IB assays were conducted to analyze the ubiquitination levels of exogenous LARS1 ubiquitination levels in 293T cells transfected with HA-Ub, Flag-LARS1, His-USP7 plasmid treated with MOTS-c. (C) LARS1-bound proteins in A2780 and SKOV3 cells were immunoprecipitated after MOTS-c and MG132 treatment, and the lysates were subjected to protein quantification and then performed Western blot analysis. (D) Western blot assay was conducted to analyze the effect of MOTS-c (30 μM) treatment on USP7 expression in A2780 and SKOV3 cells.


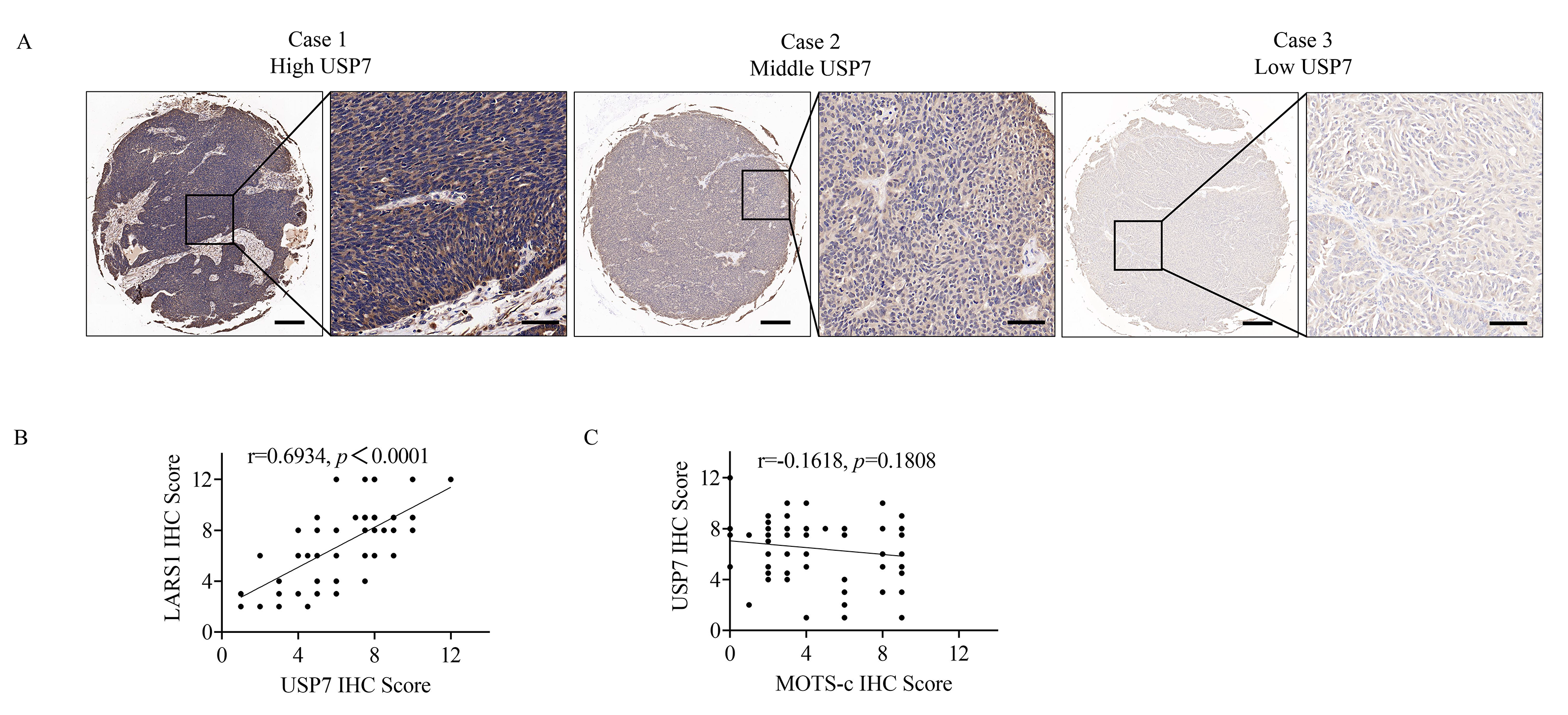


**Figure S5.** (A) IHC staining of USP7 in OC tissues, paraffin section was derived from serial sections of the same wax block. (B) Correlation analysis of USP7 and LARS1 expression in OC tissues. (C) Correlation analysis of USP7 and MOTS-c expression in OC tissues.


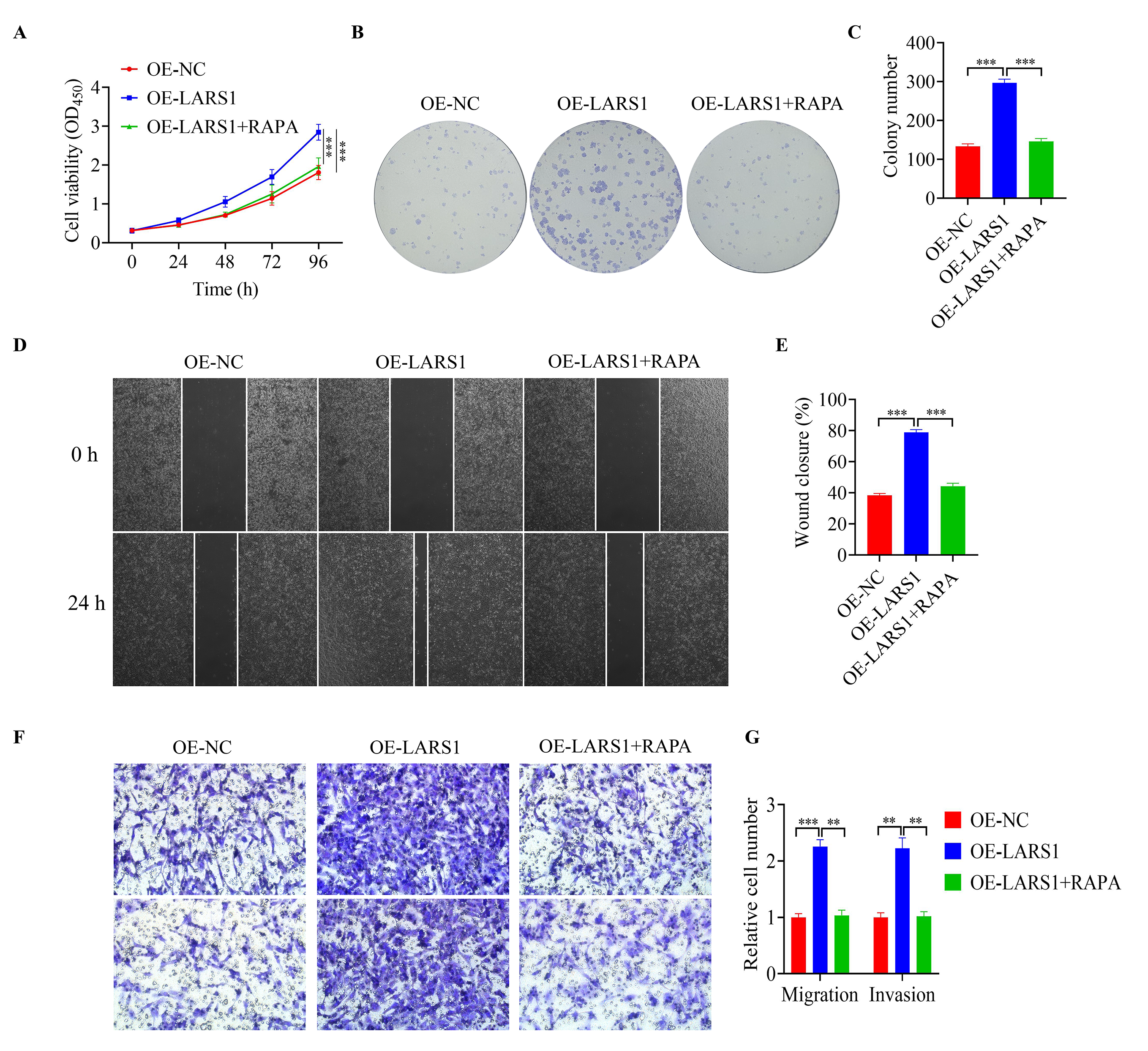


**Figure S6.** SKOV3 cells transfected with LARS1 overexpressing plasmids (OE-LARS1) or empty vector plasmids (OE-NC), OE-LARS1 cells were treated with or without rapamycin (RAPA). Three groups of cells were subjected to (A) CCK8, (B, C) colony formation, (D, E) wound healing and (F, G) transwell assays, respectively. Data are presented as the mean±SEM, **p<0.01, ***p<0.001.
